# Transposable element landscapes in aging *Drosophila*

**DOI:** 10.1371/journal.pgen.1010024

**Published:** 2022-03-03

**Authors:** Nachen Yang, Satyam P. Srivastav, Reazur Rahman, Qicheng Ma, Gargi Dayama, Sizheng Li, Madoka Chinen, Elissa P. Lei, Michael Rosbash, Nelson C. Lau

**Affiliations:** 1 Boston University School of Medicine, Department of Biochemistry, Boston, Massachusetts, United States of America; 2 Brandeis University, Department of Biology and Howard Hughes Medical Institute, Waltham, Massachusetts, United States of America; 3 Nuclear Organization and Gene Expression Section, NIDDK, NIH, Bethesda, Maryland, United States of America; 4 Boston University Genome Science Institute, Boston, Massachusetts, United States of America; University of Kansas, UNITED STATES

## Abstract

Genetic mechanisms that repress transposable elements (TEs) in young animals decline during aging, as reflected by increased TE expression in aged animals. Does increased TE expression during aging lead to more genomic TE copies in older animals? To address this question, we quantified TE Landscapes (TLs) via whole genome sequencing of young and aged *Drosophila* strains of wild-type and mutant backgrounds. We quantified TLs in whole flies and dissected brains and validated the feasibility of our approach in detecting new TE insertions in aging *Drosophila* genomes when small RNA and RNA interference (RNAi) pathways are compromised. We also describe improved sequencing methods to quantify extra-chromosomal DNA circles (eccDNAs) in *Drosophila* as an additional source of TE copies that accumulate during aging. Lastly, to combat the natural progression of aging-associated TE expression, we show that knocking down *PAF1*, a conserved transcription elongation factor that antagonizes RNAi pathways, may bolster suppression of TEs during aging and extend lifespan. Our study suggests that in addition to a possible influence by different genetic backgrounds, small RNA and RNAi mechanisms may mitigate genomic TL expansion despite the increase in TE transcripts during aging.

## Author summary

Transposable elements, also called transposons, are genetic parasites found in all animal genomes. Normally, transposons are compacted away in silent chromatin in young animals. But, as animals age and transposon-silencing defense mechanisms break down, transposon RNAs accumulate to significant levels in old animals like fruit flies. An open question is whether the increased levels of transposon RNAs in older animals also correspond to increased genomic copies of transposons. This study approached this question by sequencing the whole genomes of young and old wild-type and mutant flies lacking a functional RNA interference (RNAi) pathway, which naturally silences transposon RNAs. Although the wild-type flies with intact RNAi activity had little new accumulation of transposon copies, the sequencing approach was able to detect several transposon accumulation occurrences in some RNAi mutants. In addition, we found that some fly transposon families can also accumulate as extra-chromosomal circular DNA copies. Lastly, we showed that genetically augmenting the expression of RNAi factors can counteract the rising transposon RNA levels in aging and promote longevity. This study improves our understanding of the animal host genome relationship with transposons during natural aging processes.

## Introduction

All animal genomes carry the genetic burden of a sizeable reservoir of parasitic elements called transposons or transposable elements (TEs). This TE burden can range from the extreme >70% proportion of the axolotl genome [1,2] to >50% in the human genome [3] to >10% in the *Drosophila melanogaster* genome [4,5]. TEs are selfish invaders of animal genomes with some potential for stimulating more rapid gene regulatory innovations like serving as novel enhancers [6], but more frequently are detrimental to animal fitness when they insert into and disrupt expression of important genes [7]. Therefore, conserved chromatin regulation and RNA-interference (RNAi) pathways must silence TEs to ensure fertility and animal health. However, these genomic defense mechanisms also weaken during animal aging concomitant with observable decreases in genomic integrity in aging cells. This phenomenon has been articulated in the hypothesis of TEs impacting aging as proposed by Gorbunova et al [8], where the decline of genome homeostasis during aging may unleash the detrimental effects of reanimating TE activity.

Initial support for this hypothesis in the model organism *D*. *melanogaster* came from studies of TE expression increasing in aging flies [9–12]. For example, mutants in chromatin silencing factors and RNAi pathway genes which repress TEs have reduced lifespans [9,10,13–16], whereas dietary restriction and overexpressing the RNAi and chromatin factors can limit TE expression and promote longevity [13,14]. Neurodegeneration modeled in aging flies through overexpressing aggregating proteins like TDP-43 and TAU also leads to elevated TE expression [17–20]. Additionally, there is evidence of a somatic population of Piwi proteins which can serve an additional TE defense mechanism that when mutated leads to shorter lifespan and loss of stem cell maintenance [13,16,21–23].

Beyond flies, mammals also must repress TEs for critical development of germ cells, embryos and neurons. Mammals have a complex, interconnected network of silencing pathways like the axis of SETDB1 [24,25], KAP1 [26–28] and the HUSH complex [29–32]; and its cooperation with histone deacetylases like SIRT6 [33,34] and histone methyltransferases like Suv39h1 and G9A [35–37]. In addition, there are DNA methyltransferases that genetically interact with the piRNA pathway to target TEs for chromatin silencing in mammalian germ cells [38–44]. Although >45% of mammalian genomes are comprised of many TE repeats, the vast majority are inactive with mainly *LINE-1/L1* implicated in somatic genome mosaicism in developing brains and individual neurons [45–53]. *LINE-1/L1* is linked to deleterious novel mutations in tumors and they are activated in cell culture models of cellular senescence [54–59]. Although TE control is clearly important to mammalian health, the large genome sizes and longer lifespans hamper comprehensive assessments of mammalian TLs during aging.

Therefore, in this study we leveraged *Drosophila’s* rapid aging, its compact genome and powerful genetic tools as significant advantages for characterizing how TLs may change during normal animal aging. An important goal of our study is to address the debate of whether TLs quantitated from Whole Genome Sequencing (WGS) of *Drosophila* genomes represent true gains in TE genomic load [60]. One bioinformatics program called TEMP [61] has been used extensively in determining TE insertions from *Drosophila* WGS [62,63] but its capacity to distinguish bona fide TE insertions from potential library sequencing artifacts has been re-examined [60]. Noting the high degree of variability in TE insertion calls from various bioinformatics programs applied to *Drosophila* WGS data [64], we therefore developed our own program called the Transposon Insertion & Depletion AnaLyzer (TIDAL) to identify the tremendous diversity of TLs across various *Drosophila* strains [65]. TIDAL’s increased specificity in TE determinations comes from requiring sequencing reads mapping to both sides of genomic locus flanking the TE insertion, with a threshold minimum of 4 reads (i.e. ~2 reads on each TE insertion junction). This specificity was benchmarked against genomic PCR tests [65], and TIDAL has characterized TLs in other *Drosophila* studies of genetic factors regulating TE silencing [66,67].

In this study, we demonstrate how WGS and extrachromosomal circular DNA (eccDNA) sequencing of aged and young flies can report changes in TLs during fly aging particularly in sensitized genetic backgrounds such as in some RNAi mutants. Although TE RNA upregulation is a recurring phenotype of aging wild-type flies, we show that genomic TLs can expand in some strains defective for RNA silencing, either because of their unique genetic backgrounds or because disrupted RNA silencing now allows TEs to expand their genomic DNA (gDNA) copy numbers. We also measure genomic TLs by tissue-specific (*i*.*e*. fly brain tissues) gDNA sequencing; and eccDNA accumulation during aging of the *ISO1* strain as an additional feature of the hypothesis of TEs impacting animal aging. Lastly, we show that genetically boosting RNAi activity in aged flies via knockdown of *PAF1* can suppress TE RNAs and extend longevity. Together, these results suggest that, in addition to possible influences of genetic background, the RNAi pathway may buffer genomic alterations by the natural increase of TE RNAs during aging and suggest *PAF1* inhibition in aging animals could be a new target for genetic suppression of TE expression.

## Results

### Recurring increase of TE RNA expression during fly aging

**Fig 1:**
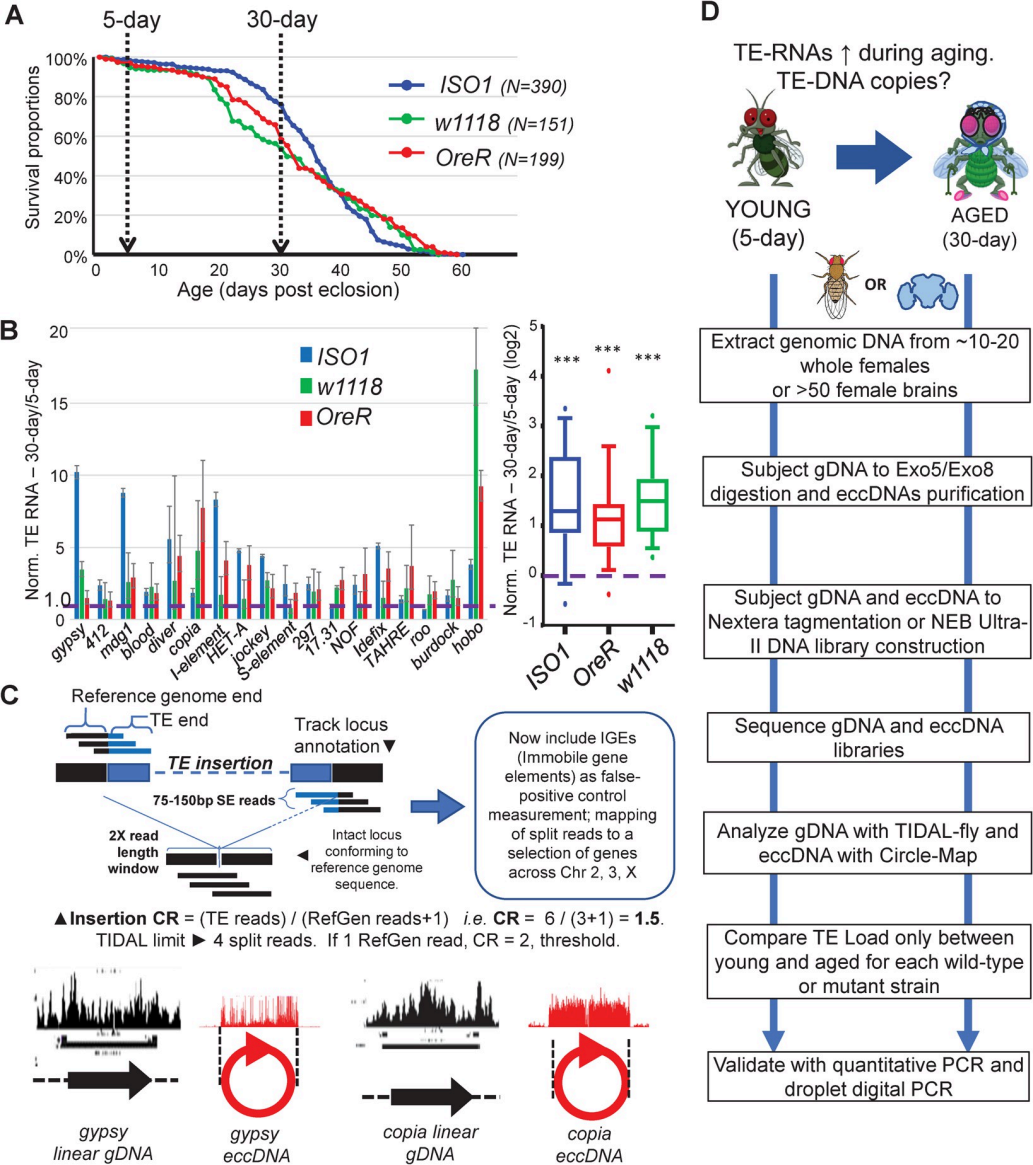
Overview of study to examine whether TE-DNA copy numbers change during fly aging. (A) Survival curves of the three wild-type (WT) fly strains carried out in this study, indicating the selection of 30-day adults as a representative timepoint of aging onset (B) Validation of TE transcript expression increases during fly aging through RT-qPCR of TE RNAs normalized to *Rp49* transcripts from whole female fly bodies. Error bars are propagated standard deviations of delta-CT values from three replicates. Boxplot on the right summarizes the data on the left plot and tests the statistical significance of TE RNA up-regulation by the Wilcoxon test in all three WT strains, *p*-value<0.0001. (C) Overview of TE detection strategy from Whole Genome Sequencing (WGS) data using updated TIDAL and extra-chromosomal circular DNA (eccDNAs) detection scripts. The coverage plots for WGS and eccDNA data are illustrated in black diagrams and red diagrams, respectively. (D) Study designed for comparing TE load between 5-day young and 30-day aged flies within each WT and mutant strain.

Although previous studies using certain control wild-type (WT) fly strains showed that TE RNAs were upregulated in aged flies [10,14], we decided to reconfirm this observation for three commonly used WT fly strains that would form the basis of this study. Using our lab’s standard rearing conditions, we first determined the aging curves for the *ISO1* strain used for the *D*. *melanogaster* reference genome sequence [4], an isogenic *w1118* strain that is a common background strain in genetic studies [68], and the *Oregon-R* strain used in a series of functional genomics datasets [69]. We established that these three strains displayed lifespans typical of other WT fly strains (**Fig 1A**).

We then followed the experimental convention of other studies [10,14] to standardize the comparison of 30-day aged adults versus 5-day young adults, and we performed quantitative RT-PCR on a panel of TEs from total RNAs from females (Fig 1B). We replicated many examples of TE RNAs being upregulated in the aged WT flies’ whole bodies but noticed variability in which specific TE families were the most significantly upregulated during aging. For example, *gypsy*, *mdg1*, and *I-element* were upregulated at the RNA level in *ISO1* aged flies, while *copia* and *1731* RNAs were upregulated in *w1118* and *OreR* (Wilcoxon rank sum test, *p*<0.001, Fig 1B). This variability may reflect the inherently distinct TLs between these three strains [65], but the trend holds true that WT adult flies recurringly experience increased TE expression during aging.

Only one recent study we are aware of assessed TLs during fly aging by WGS of enriched αβ-Kenyon Cell neurons [60] and which argued that various pitfalls obscured the ability to observe TL increases during fly aging. For example, the study itself discussed that Multiple Displacement Amplification (MDA) required to amplify the minute amount of neuronal gDNA prior to Illumina library construction could contribute to artifactual chimeric molecules that represent false positive TE insertions [60]. Therefore, our more comprehensive effort to examine TLs through direct WGS should add valuable insight to this question.

When determining TL from *Drosophila* WGS datasets, we first considered how two different TE-insertion discovery programs, TEMP [61] and TIDAL [65] can each yield different results from analyses of the same dataset (**S1 Fig**). Balancing sensitivity against specificity, TIDAL has similar trends as TEMP in revealing the diversity of TLs amongst *Drosophila* samples (S1B Fig) and both are effective at calling germline insertions, but TIDAL avoids false positive predictions that others have contended as somatic TE insertions by requiring read support on both sides of the TE insertion junction (Fig 1C versus S1C Fig, [60]). TIDAL handles this issue differently by computing a Coverage Ratio (CR) score for each TE insertion from pooled sequencing of a small group of flies (Fig 1C), where TE insertion reads are divided by reference genome mapping reads and a pseudocount of 1; such as a CR of 2 that we used as an arbitrary cutoff for indicating deep penetrance of a TE insertion at a given insertion locus [65].

### Measuring TE landscapes (TL) by direct WGS and the TIDAL program

To meaningfully compare TL changes during a single generation of aging flies from WGS and to avoid the genomic complications of normalizing against Y-chromosome reads that are exceptionally dense with repeats [70], we only compared samples from within the same strain in small numbers of young versus aged whole female adult flies or female brains (Fig 1D). In our process we extracted a set amount of genomic DNA from 10 flies that allowed for reproducible WGS library construction without requiring MDA or other total DNA amplification methods before library preparation. We then sequenced on the Illumina platform each fly strains’ bulk gDNA library to a minimum >~30 million 75-bp reads for >~16X fold genomic coverage of the version Dm6 genome assembly (**S1 Table**). Each library was analyzed identically with the TIDAL program [65] and new TE insertions were counted individually and then normalized against the reads per million measurement to account for sequencing depth differences.

**Fig 2:**
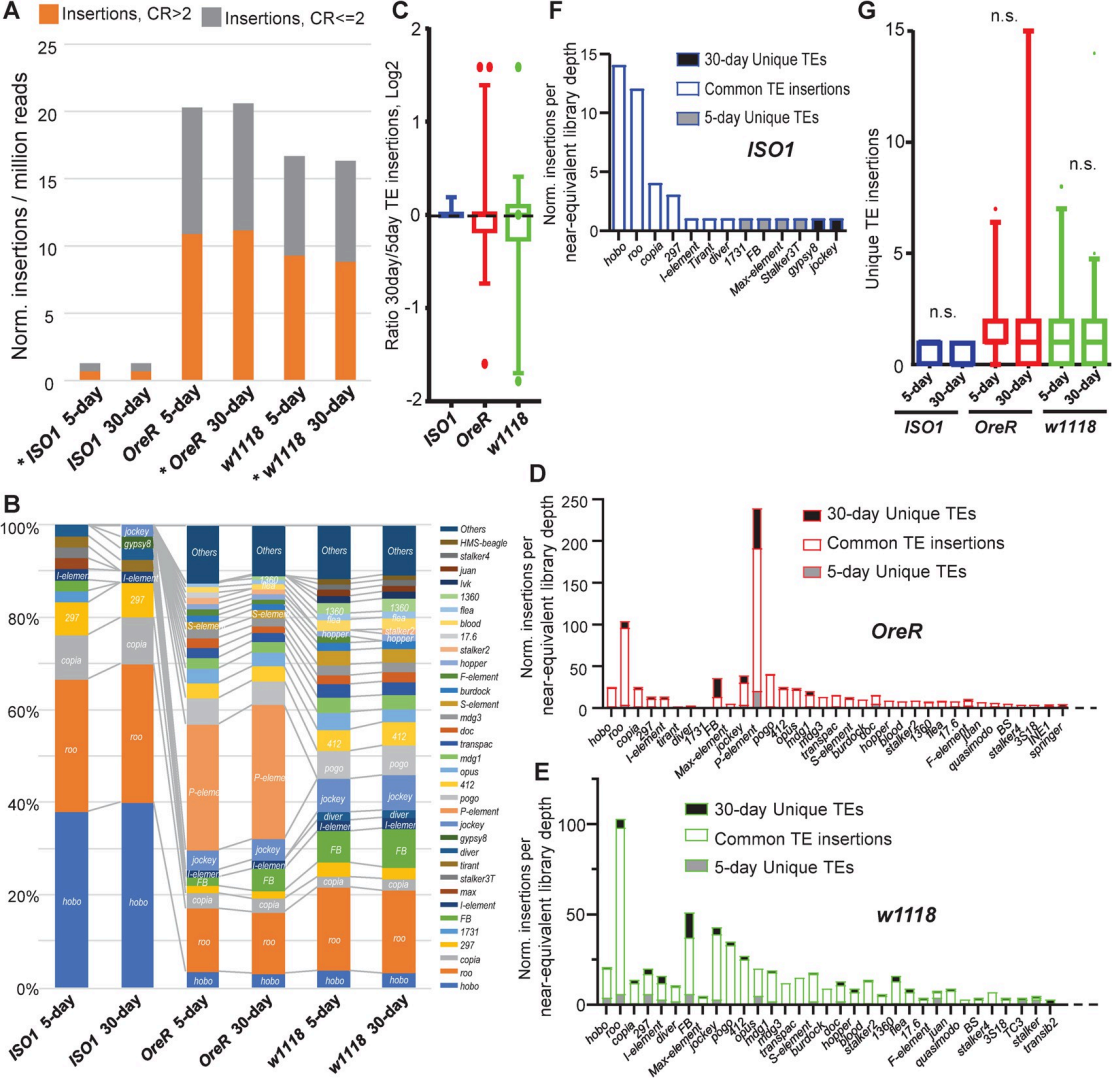
WGS analysis of TE insertion numbers between 5-day young versus 30-day aged wild-type fly strains. (A) Quantification of new TE insertions as compared to the reference genome using the TIDAL-fly program. Categories of total TE insertions broken by the Coverage Ratios (CR) of CR>2 and CR< = 2. (B) Within each strain, TE families’ percentages are ordered by the color legend. (C) Ratios of the 30-day versus 5-day of normalized TE insertions from panels D-F. (D-F) Number of unique TE insertions (filled bars) present in 5-day and 30-day relative to common insertions present in both samples (open bar) of *OreR*, *w1118* and *ISO1* fly strains. These panels display only the TE families that were detected by TIDAL be at least 1% of total number of TE families (*i*.*e*. all the TEs not lumped into the “Others” category of Fig 2B). (G) Boxplot for conducting paired Wilcoxon tests does not show statistical significance for the gain of unique TE insertions in aging WT flies. n.s. = not statistically significant.

In developing our methodology to examine fly TLs during aging, we recall our previous study showing that each fly strain’s unique TLs depends on how inherently distinct its genetic background is from the reference genome strain *ISO1* [65]. Therefore, it was expected that new TE insertions quantified and normalized against each library’s sequencing depth would yield the lowest numbers for *ISO1* and the most TE insertion differences in *w1118* and *OreR* (**Fig 2A**). In order to make equivalent TL comparisons between young and aged fly libraries, the deeper WGS library was downsampled to the sequencing depth where mapped reads were approximately the same as the lower cognate library before TIDAL analysis. We then assessed how reproducible were TL determination with TIDAL by conducting 100 randomized permutations from 50% downsampled *OreR* 5-day libraries to bootstrap TE insertion number calls (**Figs 2B, 2C and S2A**). The cumulative average number and standard deviation of TE insertion calls after 100 permutations were already reached by 20 permutations, so to economize the demanding computational resources, we applied a 20-permutation bootstrap to all the other WGS libraries reported in the main figures of this study to provide narrow 95% confidence intervals for TL determinations by TIDAL (S2D, S2E and S2F Fig). Finally, we re-analyzed the WGS datasets from [60] with TIDAL, and found that even at the single fly or single neuron level, it is challenging to detect significant TL changes in wild types (S2H and S2I Fig).

As expected, each of these WT flies TLs displayed completely distinct compositions of new insertions of TE families relative to the Dm6 reference genome sequence (Fig 2B), such as a larger proportion of *hobo* TEs in *ISO1*, major infiltration of *P-elements* in *OreR*, and several more *FB*, *pogo* and *412* TEs in *w1118*. Focusing on the ratio of 30-day to 5-day insertions for the specific TE families making up the bulk of these strains TLs, we could observe most of the TEs remained unchanged during aging whilst a few TEs have some change in copy numbers during aging (outlier dots in Fig 2C representing a few TE families). This was reflected at the total TL level with modest or few TE insertions in 30-day aged *ISO1* flies versus 5-day young flies, with also perplexing total decreases in *w1118* flies (Fig 2A). Since *OreR* and *w1118* have distinct genetic backgrounds from the *ISO1* reference genome, we further classified the total TE insertions detected by TIDAL to differentiate the “unique” from “common” TE insertions by comparing the genomic location of TE insertions between each 5-day and 30-day sample pairs. When insertions with the same genomic locus are present in both 5-day and 30-day samples, these are noted as “common insertions”; and those TE loci that are present only in one condition but not the other are classified as “unique insertions”. Although the vast majority of the TE insertions were commonly detected by TIDAL in both 5-day and 30-day *w1118* and *OreR* flies (Fig 2D and 2E), there were more TE insertions only detected in these 30-day aged flies than the 5-day young fly genomes. Only a few *hobo* insertions were also only seen in 5-day young *ISO1* flies and were no longer detected in 30-day aged flies (Fig 2F). This observation can be explained by this analysis that only focuses on TE insertion counts as quantile samplings of reads discordant from the reference genome.

New somatic transposition event in a small subset of cells could be overshadowed by a background of unmodified reference sequences and could explain a TE with a low CR score that is sampled in 5-day fly gDNA sample but then missed in the 30-day sample. This is a known limitation of the WGS approach and sacrificing sensitivity to improve specificity in the original TIDAL program [65], resulting in insufficient statistical significance to detect TL increases in WT whole flies (Fig 2G). However, we could dismiss the concern of possible skews in chromosomal structures amongst the fly genomes sequenced in this study by showing predominantly diploid sequence coverage in the FREEC plots that TIDAL automatically generates (**S3 Fig**) [65].

To tackle the potential limitation of missing reads unmappable to the euchromatic genome sequence, we updated TIDAL to also map to *Drosophila* TE families consensus sequence coverage, and added arbitrarily-selected protein coding genes, analogous to the modification to TEMP to track protein-coding genes as Immobile Gene Elements (IGEs) [60]. We gauged a relatively low average rate of ~12% of IGEs across the libraries sequenced as defined by percentage of false positive TIDAL counts from IGE versus the total number of TIDAL counts [TE+IGE] (S1 Table), whereas these protein coding genes sequencing coverage generally also remained stable between 5-day young and 30-day aged flies (**S4A Fig**). Tracking TE consensus sequence coverage has the advantage of accounting for all accumulating TE sequences in both the mappable and unassembled and ambiguous-mapping regions of the genome. With this analysis approach, we could detect some change of total TE sequence coverage in *OreR* and *w1118* 30-day aged flies versus 5-day young flies (S4A and S4B Fig, *p-*value <0.01, Wilcoxon rank-sum tests) whilst our stable protein-coding gene coverage control for these two strains remains the same (S4A and S4B Fig). For *ISO1*, the total TE sequence coverage between 5-day versus 30-day remains the same however there is a slight reduction of protein coding genes coverage in 30-day aged flies (*p*-value<0.001). However, with these being single-batch sequencing runs, the caveat of more future sequencing experiments may be needed to bolster this result. In addition, both this coverage analysis and the quantile insertions analysis cannot discriminate between a full-length or truncated TE sequence, which we have noted in *P-elements* that can have critically variable transposition activities [71].

### Resolving and validating our approaches measuring TE landscapes with RNAi mutants

This unresolved genomics challenge of using short read WGS data for analyzing TE sequences coverage also extends to some limitations in using droplet digital PCR (ddPCR) to precisely quantify genomic TE copies for only the isoforms covered by the short ddPCR amplicons [45] and little change is detected by digital PCR as well (S4C Fig). In questioning the accuracy of this ddPCR assay in absolute quantification of TE copies, we compared ddPCR results on *P-elements* and *I-elements* versus WGS and TIDAL determinations in two other directly matched gDNA samples (**S5A and S5B Fig**). The ddPCR copy number measurements were very similar to the WGS and TIDAL determinations, indicating both methodologies are consistent with each other in the quantifications. Furthermore, we replicated a previously reported genetic cross [72] that in one format triggers a large burst of *I-element* transposition in the embryos but in a second format maintains *I-element* silencing (S5C Fig). We reanalyzed the WGS datasets from [72] with TIDAL reporting 505 new *I-element* copies versus the 3732 insertions called by TEMP in that study, with our ddPCR results leaning closer to the TIDAL count (1590 copies, S5D and S5E Fig). These data reaffirm the findings from [72] that the oocyte is the critical battleground between the host and the selfish genetic element.

**Fig 3:**
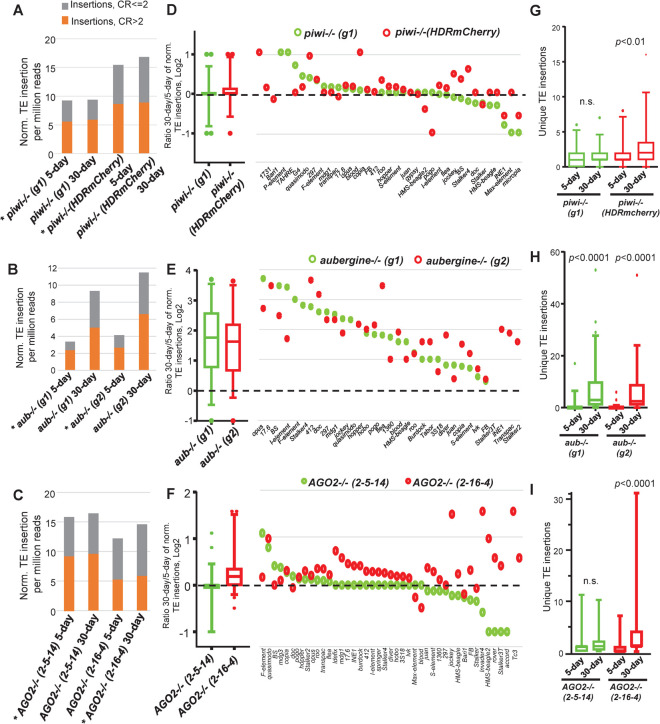
WGS analysis of TE insertion numbers between 5-day young versus 30-day aged RNAi mutant fly strains. (A-C) Quantification of new TE insertions as compared to the reference genome using the TIDAL program. Categories of total TE insertions broken by the coverage ratios (CR) of CR>2 and CR< = 2. Asterisks mark the library that was downsampled to the equivalent depth of the cognate comparison library. (D-F) Left, 5–95 percentile boxplots of ratios of the 30-day versus 5-day of normalized TE insertions from panels A-C. Right, dot plots display only the TE families with significant changes between the two timepoints in at least one of the mutant strains. (G-I) Boxplots for conducting paired Wilcoxon tests for statistical significance in the gain of unique TE insertions in aging RNAi mutant flies. *p*-value from Wilcoxon tests, n.s. = not statistically significant.

To explain why aging-associated TL changes seemed muted or were challenging to detect in WT fly strains, we considered two competing hypotheses: (1) non-penetrant TE insertions are masked by multiple unmodified genomic loci within the pools of sequences imposing limitations in WGS and TIDAL analysis versus (2) WT flies retain RNAi defenses like TE-targeting siRNAs [21,22,73–75] and piRNAs [13,76–78] to restrain further TE RNAs accumulating and contributing to genomic transposition. To test these hypotheses, we collected the same 5-day and 30-day aging cohorts from three sets of different mutants in the two main arms of the RNAi pathway in *Drosophila* (**Fig 3**). We analyzed two independent mutants each in the *piwi*, *aubergine*, *and AGO2* genes and conducted the same whole flies WGS and TIDAL analysis as the WT strains.

Both *aubergine* (*aub*) mutant alleles displayed the greatest number of TE copy increases during aging (Fig 3B, 3E and 3H and S1 Table), with both high penetrance insertions (orange bars, CR>2) as well as less penetrant insertions (grey bars, CR< = 2). However, the degree of aged associated total TE copy increases in *piwi and AGO2 mutants* was more variable between alleles (Fig 3A and 3C). Although various individual TE families appeared to increase in copy number during aging (Fig 3D, 3E and 3F), when the entire TL was considered, then only one of the two alleles displayed a statistically significant increase of TE insertions unique to the aged samples (Fig 3G, 3H and 3I). Although we attribute these TL changes to the severe lack of RNAi suppression, we cannot rule out a potential contribution by the unique genetic background effects in these denoted alleles.

### Measuring TL changes in fly brains during aging

**Fig 4:**
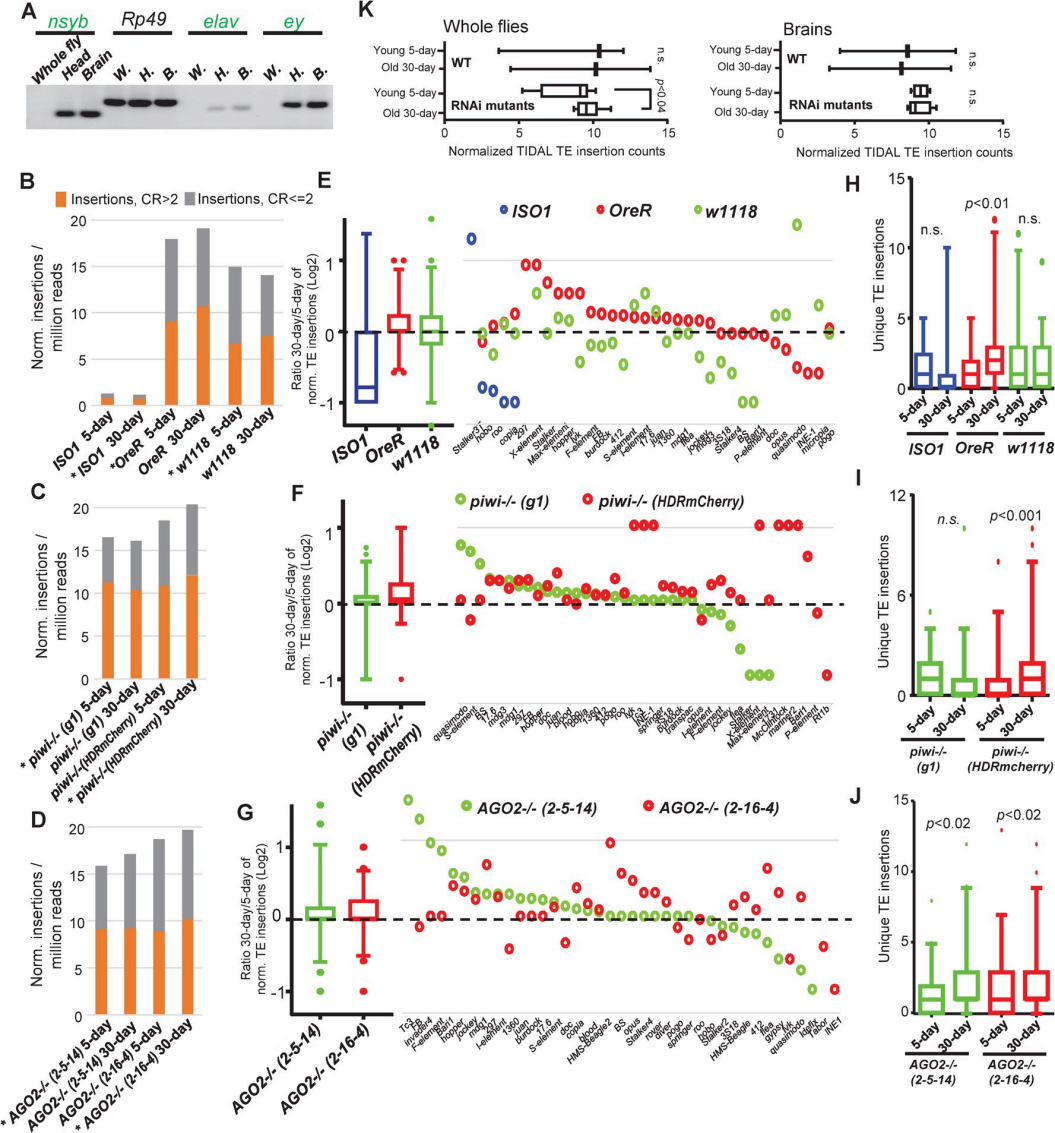
Aging-associated TE landscapes in fly brains of WT and RNAi mutant strains. (A) Validation of fly brain dissections by RT-PCR of brain-specific gene expression. TIDAL analysis of WGS for new TE insertions in the brains of (B) Wild-type (WT) strains, (C) *piwi* mutants, and (D) *AGO2* mutants. The bar graphs on the left represent categories of total TE insertions broken by the coverage ratios (CR) of CR>2 and CR< = 2. Asterisks mark the library that was downsampled to the equivalent depth of the cognate comparison library. (E-G) Left, 5–95 percentile boxplots of ratios of the 30-day versus 5-day of normalized TE insertions from panels B-D. Right dot plots display only the TE families with significant changes between the two timepoints in at least one of the mutant strains. (H-J) Boxplots for conducting paired Wilcoxon tests for statistical significance in the gain of unique TE insertions in aging RNAi mutant fly brains. (K) Boxplots of the grouped differences of paired TE insertion counts between 30-day versus 5-day amongst whole flies and fly brain WGS libraries between WT and RNAi mutants. *p*-value from a one-tailed Wilcoxon rank-sum tests, n.s. = not statistically significant. Details of the samples and values in used to build these plots are in S1 Table, which only include the newer CRISPR/genome edited mutants versus the wild-type strains, as discussed in the main figures and discussion.

Can new TE insertions also be detected in specific tissues where cells that are more permanent and not turned over as frequently, such as the brain? For example, in mammalian neurons, the most active TE *LINE-1/L1* has been implicated in transposing relatively frequently during development to give rise to genomic mosaicism in the brain [45–53]. Given the caveats of having to do prior total DNA amplification from limited gDNA from fly neurons [60], we undertook WGS from at least 50 dissected female brains to provide sufficient nucleic acid for RT-PCR confirmation of neuronal gene expression and WGS of brain DNA (**Fig 4**).

We successfully generated libraries directly from brains of WT fly strains and *piwi* and *AGO2* mutants without any prior total DNA amplification, and after setting libraries to sequencing depths with equivalent mapped reads by downsampling, we could detect some increases in specific TE families from *OreR* during aging (Fig 4B and 4E). Although there may be piRNA-like small RNAs and *piwi* expression in fly heads [13,21,22], we detected increases in TLs in the *piwi(HDRmCherry)* mutant’s brains that were similar in magnitude to the WT *OreR* strain (Fig 4C and 4F, *p*<0.01 and *p*<0.001 respectively). Additional increases in total TLs were also detectable in the *AGO2* mutants’ brains (Fig 4D, 4G and 4J), but with so few brain samples and rather low increase of TEs in the *piwi(g1)* mutant, this rendered the aggregate test of all the *RNAi* mutants’ brains as not statistically significant enough to ascribe an aging-related TE landscape increase like in whole flies (Fig 4K).

Although we cannot rule out the possible influence of unique genetic backgrounds in these RNAi mutants, we attribute the different TL changes with different penetrance levels to the separate germline versus somatic roles of each RNAi mutant gene. Because both *aubergine* and one of the *AGO2* and *piwi* mutants displayed a higher total TE number in aged flies compared to young flies (Fig 3), we suggest that RNAi can normally repress TE transcripts and might also limit genomic damage during aging. Furthermore, our data show that a direct WGS approach that obviates prior whole DNA amplification can still detect TL changes, albeit only in sensitized genetic backgrounds like the RNAi mutants such as in *aubergine* where the effects may come from ovaries in particular (Fig 3B, 3E and 3H).

### Extra-chromosomal circular DNAs (eccDNAs) as an additional genomic cache of increasing TE sequences

In normal and diseased animal cells, there is a cache of eccDNAs that has recently been explored by deep sequencing of DNA that is resistant to extensive exonuclease digestion [79–82]. In certain tumor samples, eccDNAs are implicated in rapid copy-number expansion of oncogenes [83], while ectopic accumulation of DNA in the cytoplasm of senescing cells might trigger aging-associated inflammation responses [84]. Several earlier studies had also found evidence of eccDNAs in *Drosophila*, with the *copia* TE as a prominent example accumulating in certain strains [85–89]. Lastly, eccDNA enriched in TE sequences and other repeats were detected in normal plants and gDNA of human tissues [79,90], which in both of these studies required total DNA amplification prior to library construction to enrich the surviving eccDNAs after exonuclease digestion.

**Fig 5:**
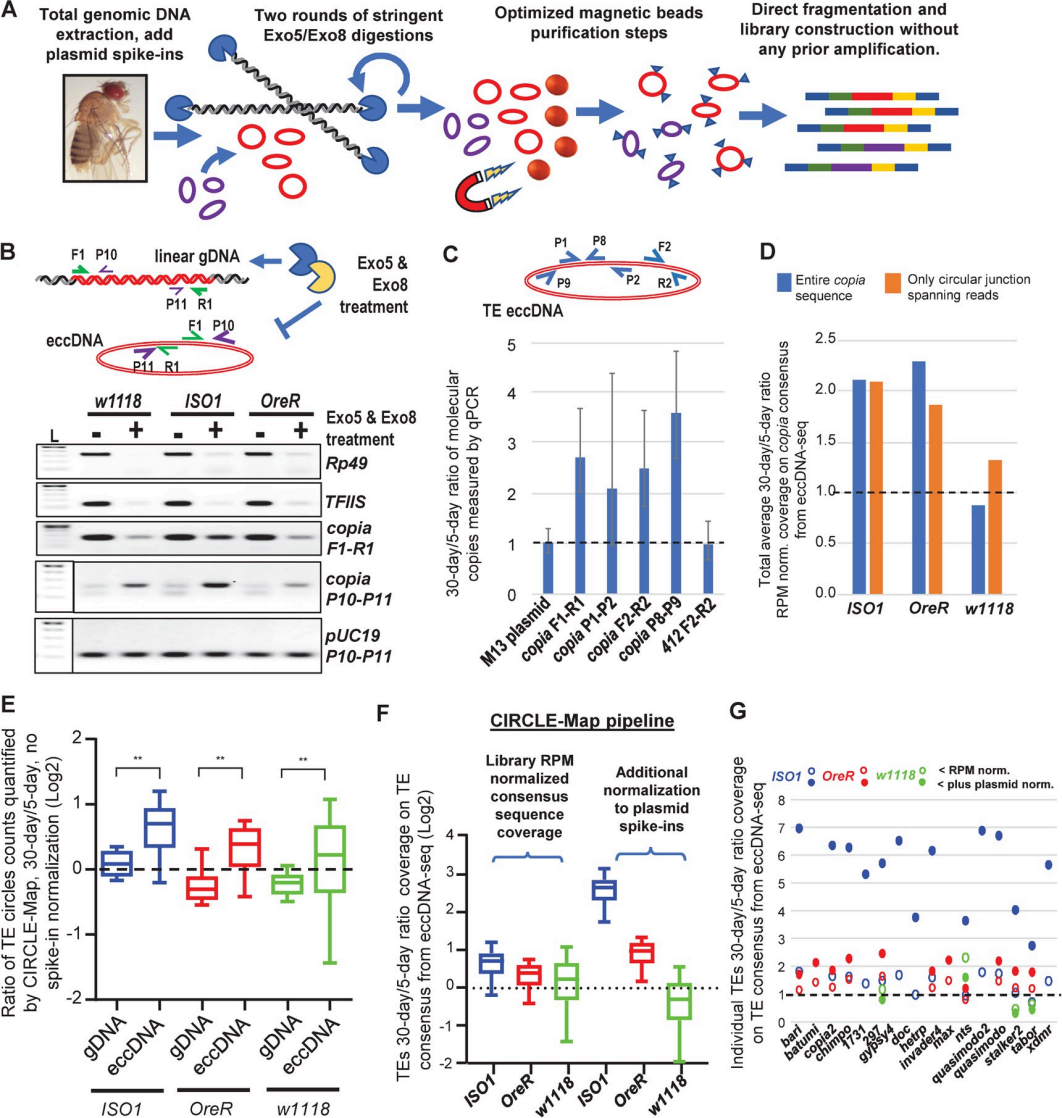
Aging *Drosophila* display increases in TEs existing as extra-chromosomal circular DNA (eccDNA). (A) Diagram of methodology to enrich and purify eccDNAs for direct library construction and sequencing without requiring prior amplification. (B) Genomic PCR from WT flies demonstrating the depletion of linear gDNA and enrichment of eccDNA with TE sequences during exonucleases treatments. The diagram above explains configuration of PCR primers. L = DNA ladder. (C) qPCR validation of spike-in plasmids and *copia* eccDNA after exonucleases treatments of *ISO1* gDNA from young versus aged adult flies. The diagram above explains the configuration of PCR primers. (D) Ratio of the read coverage just across the *copia* consensus sequence comparing young versus aged flies. (E) Comparison of the ratios of 30-day to 5-day CIRCLE-Map counts of TE circles between total gDNA libraries and eccDNA-enriched libraries without plasmid spike-in normalization. (F) Box plots of 30-day/5-day ratios of read coverage for eccDNA TE sequences rated by the CIRCLE-Map pipeline with a positive “circle score” [79] comparing to additional normalization to plasmid spike in. (G) Dot graph highlighting specific TE eccDNAs whose 30-day/5-day sequencing ratios are normalized to library RPMs (outlined dots) and to the plasmid spike-ins (filled-in dots) from a subset of (F) for TE families that had significant “circle score” >50.

We investigated eccDNAs in *Drosophila* by optimizing our own method to purify enough eccDNAs to directly generate libraries for deep sequencing without requiring prior total DNA amplification (**Fig 5A**). Furthermore, we used spike-ins of cloning-vector plasmid DNAs into gDNA preparations and magnetic beads for improved recovery and quantitation of eccDNAs for comparing between different samples. To confirm that eccDNA was recovered after two rounds of Exo5 and Exo8 exonuclease digestion steps which only degrade linear but not circular DNA, we conducted PCR with standard primers amplifying linear genes and TEs (F1-R1 primer pairs, **S2 Table**), and outward-facing primers that either generate an amplicon from a TE eccDNA or tandem genomic copies of the same TE (P10-P11 primer pairs) (Fig 5B). Linear gene amplicons were significantly depleted after exonuclease digestions, while the amplicon for the spike-in plasmid was maintained. Linear TE amplicons were also reduced while eccDNA-targeted amplicons for the *copia* TE was resilient against the exonuclease treatment. Some other TE amplicons with outward-facing primers that were reduced after exonuclease treatment may reflect more tandem copies of these TEs.

Since the regular PCR amplicons for the *copia* eccDNA were readily apparent in WT strains (Fig 5B), we used qPCR to quantify the changes and show that *copia* eccDNA copies were increased >~2-fold in 30-day aged flies compared to 5-day young flies (Fig 5C). This result motivated us to deeply sequence short read libraries generated directly from those eccDNA-enriched samples which did not undergo any total DNA amplification (**S3 Table**). After confirming from a genome browser view the enrichment of *copia*, *gypsy*, satellite repeats and mitochrondrial eccDNA enrichment from these libraries (**S6A–S6D Fig**), we adapted the TIDAL scripts of mapping reads to the TE families consensus sequences to measure sequencing coverage as well as circular junction spanning reads against *copia* and observed an aging-associated increase in *copia* eccDNA that was consistent with our qPCR results (Fig 5D). We also applied this custom eccDNA quantitation pipeline to all the other *Drosophila* TEs as well as adapting the CIRCLE-Map pipeline previously used to measure mammalian eccDNAs [79] to the *Drosophila* TEs. We then normalized the ratios of the eccDNA-TE counts between 30-day aged and 5-day young flies (Fig 5E). Although the CIRCLE-Map pipeline was more sophisticated at providing a significance “circle score” that we set the cutoff to be >50, our custom eccDNA quantitation pipeline’s results were notably consistent in showing overall that most eccDNAs as TEs were increasing in the libraries of 30-day aged flies (Figs 5F, 5G, S6G and S6H). However, the additional normalization to the plasmid spike-ins were also informative in moderating eccDNA levels in *w1118* while reaffirming the TE eccDNA increases in *OreR* and *ISO1* (Fig 5F and 5G). The plasmid spike-ins controlled for variations in extraction procedures and linker addition steps between samples during the eccDNA library construction. Thus, while *ISO1* TLs did not change much at the chromosomal level during aging, *ISO1* TE copy numbers may instead increase through eccDNA accumulation. Intriguingly, the *ISO1* strain showed the least chromosomal TL changes yet exhibited the greatest increase in TE-eccDNAs in the whole flies, while the *OreR* and *w1118* strains also showed evidence of TE-eccDNAs accumulating in the brain after normalization to plasmid spike-ins (S6E Fig).

We have confidence in our quantification of TE-eccDNA increases during fly aging because of four reasons. (1) The increase in TE-eccDNAs during fly aging detected by the CIRCLE-Map program was evident in the eccDNA-enriched libraries while being clouded by background in WGS libraries from gDNA (Fig 5E). (2) There was no aging increase and low counts in the eccDNA libraries for IGEs, since these would be in linear gDNA that would be extensively degraded by the exonuclease treatments (S6F Fig). (3) The current results (Round 10) show plasmid spike-in counts as a ratio of the eccDNA library being similar to the ratio of counts for TE-eccDNA (S6I Fig), which contrasts against an earlier eccDNA sequencing run (Round 9) that we did not analyze further because of too much plasmid spike-in. (4) Mitigating concerns of pipetting variability between samples, we observed across multiple samples consistent ratios between the counts for a group of plasmids that were spiked-in prior to exonuclease treatment relative to a fifth plasmid spiked-in even earlier, prior to gDNA extraction (S6J Fig). Together, these metrics will be helpful towards evaluating the success of future eccDNA sequencing efforts, which we can envision to be adding an extra heterologous gDNA spike-in to complement the plasmid spike-in.

### Other considerations of TE landscapes in other RNAi mutants

In addition to variations in TLs between WT strains, we also observed differences in TLs between other RNAi mutants that we cannot fully explain. For example, we examined aging-associated TLs from two EMS-induced point mutants of *Dcr-2 (L811fsx)* and *Dcr-2 (R416X)* from [91]), the nuclease acting upstream of *AGO2* to generate the siRNAs from TE dsRNAs. However, there was inconsistent and contrary TL differences between young and aged *Drosophila* in these *Dcr-2* mutants whole flies and brains (**S7A and S7B Fig**) as well as in *AGO3* mutants (S7C Fig, [92]). Perhaps these sets of mutants are not as penetrant in the loss of RNAi activity such as the persistence of siRNAs in the two *Dcr2* mutants [91], these *AGO3* mutants actually do not lose fertility nor lose TE repression [92], or there might be potential off target effects contributed by different genetic backgrounds. Furthermore, the analysis of a partially rescuing *AGO2* transgene in the *AGO2 (2-5-14)* null mutant did lower the initial levels of TE insertion differences noted by TIDAL, but this partial rescue (where the AGO2 transgene did not reach wild-type expression levels) still did not prevent aging-associated TE increases (S7D Fig). Nevertheless, we propose that RNAi activity must be sustained during aging to mitigate negative effects of increased TE expression in aged flies, a phenotype that has also been frequently observed in mammals [34,55,93,94].

### Genetically enhancing RNAi counteracts TE expression during Drosophila aging

**Fig 6:**
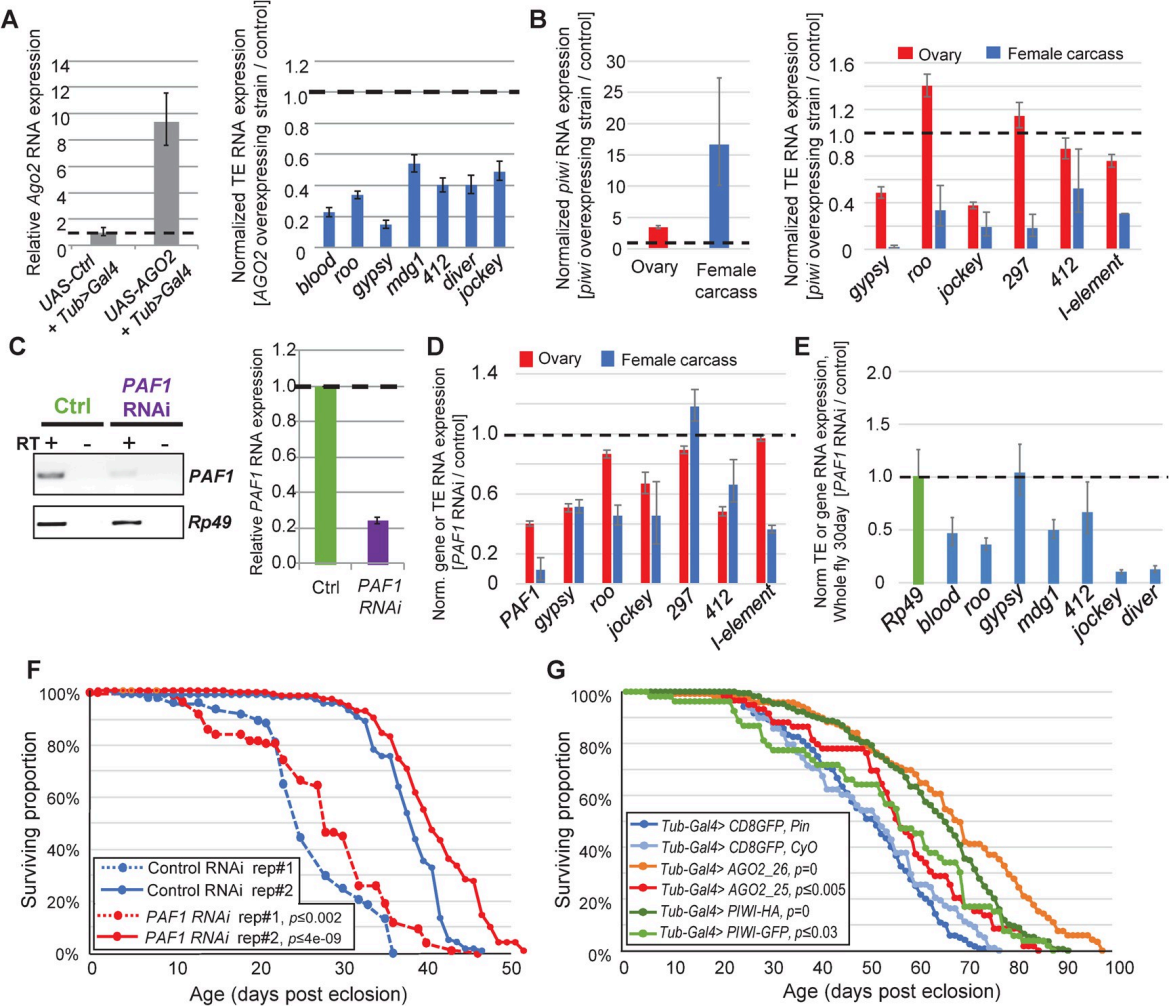
Genetic interventions of TE expression in adult *Drosophila*. (A) Overexpressing *AGO2* [*UAS-HA-AGO2/Tub>Gal4*] and (B) overexpressing PIWI [*UAS-3X-HA piwi*/+; *Tub>Gal4*/+] results in a reduction of TE RNA expression in 5-day young adult *Drosophila*. Left graphs confirm gene overexpression and right graphs detail TE RNA expression measured by RT-qPCR of the target gene compared to the *Rp49* housekeeping gene and with error bars representing propagated standard deviation of triplicate measurements. (C) Adult-specific knockdown of *PAF1* in 5-day young females qualitatively assessed in the gel (left) and RT-qPCR (middle), which reduces TE RNA expression (right) using *Tub>Gal4; PAF1 RNAi*. Examining the effect of TE RNA reduction in the *PAF1* knockdown in the ovary and carcass of 5-day flies (D) and 30-day whole flies (E). (F) Life span comparison between control versus *PAF1 RNAi* knockdown of adult female flies upon raising them at 29°C to release the Gal80^ts^ inhibitor to induce RNAi from the *Tub>GAL4*. *PAF1 RNAi* N = 112,119, *Control RNAi* N = 170,153, rep#1 and rep#2, respectively. (G) Life span comparison between strains overexpressing *AGO2* and *PIWI* and control strains *CD8GFP*. *AGO2_25*, N = 119, *AGO2_26*, N = 59, *PIWI-HA*, N = 169, *PIWI-GF*P, n = 53, *CD8GFP/+*,*CyO/+*, n = 98, *CD8GFP/+*,*Pin/+*, N = 120. *p*-values are from the log rank test calculated with the Oasis tool [123].

Although TE expression still increased in WT aging flies, we hypothesized whether endogenous RNAi pathways that still limit genomic TL increases could also be genetically enhanced to mitigate the aging-associated rise of TE RNAs. To test this hypothesis, we first used a ubiquitous *Tubulin-GAL4* driver to overexpress *AGO2* in adults, and as expected, multiple TE RNAs had lowered expression relative to the negative control (**Fig 6A**). We then used the same driver to overexpress *piwi*, and although there was likely a silencing limit to prevalent *piwi* expression in the ovary, the enhancement of *piwi* expression and TE silencing were much more apparent in the female carcass (Fig 6B). Although the status of the genetic backgrounds and TE expression levels of the parental strains in these GAL4-UAS experiments is uncertain, these data provided a proof of principal that augmenting these RNAi pathways in adults result in improvements in TE silencing.

However, inhibiting a factor that normally limits RNAi activity would be preferable from a therapeutic standpoint. Examples of endogenous negative regulation of RNAi activity include proteasome-mediated turnover of *AGO2* [95], ENRI factors that negatively regulate nuclear RNAi in nematodes [96], and the RNA exosome and *PAF1*’s transcription elongation role modulating RNAi silencing activity on TEs conserved in both fission yeast and flies [97–99]. Even though we were able to use siRNA knockdown of *PAF1* in *Drosophila OSS* cells to demonstrate enhanced TE silencing, we recognized that genetic knockdowns of this essential modulator of RNAi would also have detrimental effects on development like its requirement in ovarian development [97].

So, to circumvent developmental impacts of *PAF1* knockdown in flies, we further combined the temperature-sensitive inhibitor of GAL4 expressed from a second transgene of *Tubulin-Gal80*^*ts*^ with the *Tubulin-Gal4* driver [100]. This double-transgenic fly could then be crossed to the same *UAS-PAF1-RNAi* line so that flies can develop fully at the permissive temperature of 18°C, and after eclosion be raised at 29°C to trigger the RNAi knockdown of *PAF1* (Fig 6C). Because elevated temperature itself can affect TE silencing activity in flies [101–104], we used an *mCherry-shRNA* strain as a negative control that was also raised at 29°C at the same time as the *PAF1* knockdowns. There was appreciable enhancement of TE silencing in the whole female flies (Fig 6C) with similar levels of TE silencing enhancement between the ovaries and the soma (Fig 6D and 6E). We attribute the increased TE silencing during *PAF1* knockdown to the reduced elongation rate of TE transcripts so that RNAi factors can better engage [97] and not from a global transcription reduction because steady state levels of control gene, *TFIIs*, *AGO2* and *piwi* were not reduced by *PAF1* knockdown (**S8A Fig**).

We then measured lifespans from two cohorts of flies where *PAF1* knockdown was compared to the *mCherry-shRNA* negative control with the *Tubulin-Gal80*^*ts*^ and *Tubulin-Gal4* driver cross at 29°C (Fig 6F). Although one cohort measured at the start of the Covid-19 pandemic initially dipped at 2 weeks, the *PAF1 RNAi* knockdown flies ended up living statistically longer than the control, and this lifespan extension was more pronounced and replicated in a second cohort. To support the notion that *PAF1* knockdown was enhancing RNAi activity to extend *Drosophila* lifespan, we also observed a significant lifespan extension from multiple lines overexpressing *AGO2* and *PIWI* (Fig 6G). Although the control strain used for comparisons to *AGO2* and *PIWI* overexpression also express GFP, perceptions of GFP toxicity can be tempered by that fact that these control animals outlive all other WT strains (Fig 1A).

Since we had observed TE landscape activity in the adult fly brain (Fig 4), we also tested a brain-specific driver, *elav-GAL4*, that was effective at triggering *PAF1* knockdown and enhancing TE silencing in the 30-day aged fly brains (S8A Fig). However, this *elav-GAL4* driver that is expressed as early as during embryonic development [105] was likely also reducing *PAF1* to a point that it lowered the overall fitness relative to the control (S8A Fig). To bypass the critical embryonic developmental stages, we were able to finally recombine *Tubulin*-*Gal80*^*ts*^ with the *elav-GAL4* and confirmed neuronal-driven GFP expression after 29°C induction (S8B Fig). However, neither *PAF1* knockdown nor TE silencing enhancement could be observed initially from third-instar larvae or eclosed adult 29°C induction, although some reduced *PAF1* and TE expression was eventually observed in 30-day old adults (S8C Fig). The inclusion of *Tubulin*-*Gal80*^*ts*^ has somehow tempered the effectiveness of the *elav-GAL4-PAF1 RNAi*, so that neither developmental issues of premature *PAF1* loss nor longevity extension was seen (S8D Fig), but a final experiment using the drug RU486 to trigger *Geneswitch->elav-Gal4-PAF1-RNAi* was able to cause more widespread gene and TE expression knockdown and extend lifespan versus the ethanol control. Together, these data suggest that future spatial and temporally controlled inhibition of *PAF1* to optimal levels may be a relevant avenue of intervening with the aging-associated increase in TE expression in maturing adult animals.

## Discussion

In this study we conducted an analysis of WGS approaches towards assessing changing TLs during *Drosophila* aging, and we found that TL increases are detectable in the genomes of aging RNAi mutants, especially *aubergine*. These mutants are viable although others have shown that they have reduced longevity compared to control strains [10,13,16], and our data now confirms that unchecked elevation of TE transcripts can result in quantifiable genomic alterations in a single lifetime of flies. This result was primarily supported by the data in *aubergine* flies, suggesting that the germline is the primary location of this activation. However, it was more difficult to detect new TE insertions amongst the gDNA of WT fly strains: we had to focus the TIDAL analyses on specific TE families mobilizing into uniquely-mapping sequences and also count the coverage on TE family consensus sequences (S3 Fig). After showing that an orthogonal quantitation method like ddPCR is consistent with TIDAL’s quantitation of TE copy numbers from WGS of *P-elements* and *I-elements* (S4 Fig), our parsimonious conclusion is that despite aging-associated increases in TE expression during fly aging, the RNAi pathway may protect the fly genomes from accumulating new TE insertions, particular from the ovary which can still contribute to the overall longevity of female flies. Furthermore, our study expands the dimension of WGS of TLs by incorporating eccDNA as an *in vivo* cache of accumulating TE DNA sequences (Fig 5).

Despite the compactness and completeness of the *D*. *melanogaster* genome sequence, technical challenges still remain to fully optimize WGS approaches for quantifying TLs. For example, all current metazoan genome assemblies still suffer from large sequencing gaps in telomeric, centromeric and other repetitive regions that remain challenging to analyze. Meanwhile, long-read sequencing like Nanopore and PacBio that could close these gaps are still less economical and not as accurate as the Illumina sequencing platform [106], yet library construction methods for the Illumina platform require sufficient input material for reproducible generation of sequencing libraries. Single-cell WGS is not yet robust enough nor has total DNA amplification approaches been demonstrated to be unhampered by molecule bias, so our study required pools of genomes and non-amplified input DNA samples to reduce the prior concerns.

In retrospect, our study had to balance several competing factors such as (1) reducing to as few a number of individual animal genomes for gDNA input versus (2) generating WGS libraries that robustly sequence without prior whole DNA amplification versus (3) reducing the presence of artifactual PCR amplicons like IGE insertion events versus (4) achieving nearly equivalent sequencing depths and conditions between samples amidst inherent biochemical and bioinformatics limitations. For instance, preliminary single fly WGS libraries will require further optimization because IGE artifacts were unacceptably higher than our settled approach here to pool 10 whole flies and 50 brains of a particular strain which has the caveat of yielding differences in the normalized TE insertion count number. Future studies will be needed to improve sensitivity and accuracy of WGS analysis of *Drosophila* TE landscapes, because our current data does not capture the single-cell microscopy detection of transposon-based genetic reporters like the *gypsy-TRAP* and *gypsy*-CLEVR that can detect increased transposition activity in aging flies [13–15,18,107]. These reporters have the advantage of low cost and sensitivity of detecting small numbers of cells in a background of nonmodified cells, yet this transgenic construct is also only designed for *gypsy* to insert and activate a fluorescent protein read-out and cannot assess overall genomic TLs.

With the pleotropic effects of TE expression during aging, it remains a worthy goal to combat this potential impact such as with therapeutic approaches using reverse transcriptase inhibitors to inhibit *LINE1/L1* activity [33], while other studies showed that dietary restriction and prolonged exercise in animals can reduce aging-associated increases in TE expression [14,54,108]. Our study proposes an additional therapeutic target of augmenting the RNAi pathway’s response to TEs by inhibiting *PAF1*, which has a conserved impact on limiting RNAi from silencing TE transcripts [97,98]. Perhaps therapeutic siRNAs against *PAF1* transcripts can be hypothesized as a feed-forwarding therapeutic agent to augment RNAi activity in aging animal cells.

A final question to resolve in the future is what cascade of epigenetic and chromatin landscape changes during animal aging leads to increases in TE expression? Given the pleiotropic nature of the animal aging process, we anticipate that there will also be multiple genomic mechanisms that will vary in impact between different genetic backgrounds. For example, we describe variation amongst three WT *Drosophila* strains in the level of accumulating eccDNAs containing TE sequences (Fig 5), while others have shown increased in polyploidy in adult *Drosophila* brains [109] as well as somatic genome instability in regions of the *Drosophila* genome [110] that might contribute to changes at the level of TE consensus sequence coverages (S3B Fig). Lastly, during fly aging there are also gross-level changes in histone marks typically associated with chromatin silencing [12,14], which may precede the increase of TE expression, so the future extension of this work will be to add epigenetic and chromatin accessibility landscapes to TLs during *Drosophila* aging.

## Materials and methods

### *Drosophila* strains, genetic crosses and aging curves

All flies were raised at 25°C on standard cornmeal food. For fly aging analyses, newly eclosed female flies were harvested from bottles and mated with males for two days. These females were then divided into ~20 individuals per vial and flipped to new vials every 2–3 days to mitigate crowding stress according to this protocol [111]. Surviving flies were counted at each flip, and the percentage of cumulative survival rate at each time point was plotted against its corresponding age (date of counting subtracted by date of eclosion).

The isogenized *ISO1* fly strain for the Dm6 reference genome sequence was obtained from Susan Celniker [4]; the *w1118* is an isogenized strain and was a gift from R. Scott Hawley [68]; and the *OreR* from the ModEncode project was a gift from Terry Orr-Weaver [69]. The RNAi pathway null mutant strains *piwi-(g1)*, *aubergine-(g1)*, *aubergine-(g2)*, *AGO3-(g1)* and *AGO3-(g2)* were a gift from Julius Brennecke [92]. An additional mutant strain of *Piwi-[HDR-4xP3-mCherry]* [112] was a gift from Eric Lai. The null *AGO2* mutants deletion strains of *AGO2-[2–5–14]* and *AGO2-[2–16–4]* and *Ago2-WT-rescue* stocks were generated by CRISPR Cas9 approaches as described in [113,114]. The strains with active and inactive *I-elements* and spermless males were a gift from Zhao Zhang [72]. The *UAS-Ago2-HA* strains were a gift from Arno Muller lab [115] with two transgene insertions: *Ago2-25* on Chr3 and *Ago2-26* on Chr2. The *UASp-3xHA-Piwi* was a gift from the Ruth Lehman lab [116] and *UAST-GFP-Piwi* was obtained from Jean-Yeves Roignant as a kind gift from J. Brennecke. The *PAF1* knockdown RNAi line was obtained from the Vienna *Drosophila* Resource Center (VDRC#108826) and an *mCherry-shRNA* control line was obtained from the Harvard TRiP resource (BDSC#35785). The driver strains of *Tubulin-Gal4* and *elav-Gal4* and the *UAS-CD8GFP;Pin/CyO* stocks were gifts from Leslie Griffith [117]. The G*eneswitch->elav-Gal4* driver was a gift from the Marr Lab at Brandeis University. In addition to an early *Tubulin-Gal80*^*ts*^ strain we received from the Griffith lab, we also obtained a second *Tubulin-Gal80*^*ts*^ stock from the Bloomington *Drosophila* Stock Center (BDSC#7018) and combined with *Tubulin-Gal4* which later we molecularly validated before using it for the downstream aging experiments.

For the *Tubilin-Gal4* induced over-expression, parental cross between *Tubulin-Gal4* with the *UAS-CD8GFP;Pin/CyO* as control versus *UAS-Ago2* or *UAS-PIWI* were set at 25°C and F1 adult females of the control over-expression (*UAS-CD8GFP/+; +/Pin; Tubulin-Gal4/+* and *UAS-CD8GFP/+*,*+/CyO; Tubulin-Gal4/+*) versus *Ago2 (UAS-Ago2(26)/+;Tubulin-Gal4/+* and *UAS-Ago2(25)/Tubulin-Gal4)* overexpression or *PIWI (UASp-PIWI-3xHA/+;Tubulin-Gal4/+* and *UAST-PIWI-GFP/Tubulin-Gal4)* overexpression were collected and followed at 25°C for life span assay. For the *Tubulin>Gal4* induced knockdown, parental cross between *Tubilin-Gal80*^*ts*^*/+; Tubulin-Gal4/TM6* with *mCherry-shRNA* versus *PAF1-RNAi/CyO* were set at 25°C and F1 adult females with correct genotype were collected respectively and transferred to 29°C incubator and assayed for the entire life span assay. For the neuronal *elav-Gal4* induced knockdown, parental cross between the *elavGal4/CyO* driver with *mCherry-shRNA* versus *Paf1-RNAi/CyO* flies were set at 25°C and F1 flies with correct genotype were collected and assayed for the life span assay.

For the combinatory *elav-Gal4* with *tubulin-Gal80*^*ts*^ experiment, *elav-Gal4* was first recombined with *tubulin-Gal80*^*ts*^ (since both transgenes are located on Chr2) by screening from at least 20 individual F1 recombinants backcrossed to double balancers. Genotyping on F2 recombinants against both Gal4 and Gal80^ts^ primers confirmed the success of obtaining 1 line containing both transgenes. This recombinant line was further functionally validated by crossing with a *UAS-CD8GFP* stock, of which the F1 progenies with genotype of *UAS-CD8GFP/+; elav-Gal4*, *tub-Gal80*^*ts*^*/+* glow only after shifting to 29°C. These *elav-Gal4*, *tublin-Gal80*^*ts*^*/CyO;+/TM6* males were crossed with *mCherry-shRNA* versus *PAF1-RNAi/CyO* virgins and F1 adult females with correct genotypes were harvested and transferred to 29°C for the life span assay. Replicate 1 and 2 have identical genotype except one set (of both control versus *PAF1 RNAi*) are heterozygous of *TM6* instead of wildtype on Chr3.

For the Geneswitch assay, parental cross between *Geneswitch->elav-Gal4* and *Paf1 RNAi/CyO* were set at 25°C and 200 ul 100% EtOH versus 500 uM RU486 (dissolved in 100% EtOH) were added to fly vials with third instar crawling larval (generally at day 7 after initial cross set day). F1 adult females were collected and transferred periodically to the EtOH versus RU486 (same volume and concentration as added during the third instar larvae stage) fly food throughout the life span assay and counted until age up to 95 days.

To quantify *I-element* copies by ddPCR, fly cross schemes from [72] were replicated (S5C Fig). Parental crosses between the *w1118* strain with active *I-elements* and *w*^*k*^ strain with inactive *I-elements* were performed reciprocally to generate many virgin F1 females where one strain enables *I-element* transposition (“invaded” from *w*^*k*^ as the maternal parent) versus a control that maintains *I-element* silencing (*w1118* as the maternal parent). These F1 females were then crossed to sperm-less males that were obtained as F1 male progenies from the parental cross of *w*^*1118*^ virgin females with XY attached male. F2 oocytes were collected overnight and DNA was extracted for ddPCR against the *I-element* and *Rp49*.

### Fly brain isolation, genomic DNA extraction, WGS library construction and deep sequencing

Fly brains were dissected from at least 50 females per age group, following a procedure laid out in [118]. Eye disks and other tissues were removed from heads with forceps, and brain lobes were dissected into tubes with ice-cold PBS before freezing once at -20°C. Whole female flies and fly brains were homogenized in a standard DNA digestion buffer (1% SDS, 50 mM Tris-HCl pH 8.0, 100 mM EDTA, 100 mM NaCl, 0.5mg/ml Proteinase K) overnight at 50°C, and then extracted using standard phenol chloroform extraction, ethanol precipitation, and resuspending gDNA pellets in pure water.

WGS of whole flies began with the circa 2014 Nextera Tn5 tagmentation kit (Illumina) using an input of 50 ng gDNA and outputs were purified with AMpure XP beads (Beckman Coulter). WGS libraries were quality controlled with the high-sensitivity DNA kit on the Bioanalyzer (Agilent), selecting for size distributions of 300bp to 1kb and concentrations over 1 nM. Multiplexed libraries were sequenced on Illumina Nextseq500 high-output flow cells using 75 bp paired-end and single end reads. All WGS libraries were sequenced to a minimum depth of 30 million reads (S1 and S2 Tables). After determining that some whole fly libraries made using NEBNext Ultra-II DNA library prep kit for Illumina (NEB) were as complete and has better yields than the then discontinued Nextera kit, we completed the fly brain gDNA libraries with the NEBNext kit and sequenced them to similar depths as above.

### RNA extraction, quantitative RT-PCR, digital droplet PCR (dd-PCR) and TE copy number estimation

Total RNA was extracted from 5–10 female flies harvested at corresponding age with TRI-reagent (MRC, Inc.). Reverse transcription (RT) was performed using random primers, ProtoScript II (NEB), and 1 μg input of total RNA. Quantitative PCR (qPCR) with the Luna Sybr-Green mastermix (NEB) using primer sequences in S2 Table and 2 μL of a 1:10 dilution of the cDNA.

Relative changes in gene expression were calculated using the 2^ΔΔCt method with *Rp49* as a housekeeping gene for normalization. Briefly, the ΔCt value difference between TE target and housekeeping gene (*Rp49*) was calculated for both experiment groups (e.g. knockdown conditions) and control groups; and the difference between these two ΔCt values (dΔCt experiment- dΔCt control) was further calculated to obtain the ΔΔCt value. Relative fold change values (from experiment to control) was calculated from the exponent of 2 to the power of negative ΔΔCt value.

Droplet digital PCR (ddPCR) was conducted on a QX200 instrument with the Evagreen assay reaction (Biorad). Copy number measurements from specific TE primers (S2 Table) were normalized to *Rp49* as a diploid gene, starting first at 2 ng of gDNA as input per 20 μL ddPCR for droplet generation for most TEs. For TEs with very high copy numbers that saturate the droplets, input gDNA was diluted further into the ddPCR mix prior to droplet generation. At least 10,000 droplets were required to achieve good statistical estimation of the concentration calculated by Poisson distribution using Quantasoft Analysis Pro (Biorad). TE copy numbers per genome was determined by dividing against half of the measured *Rp49* copies.

### Extracellular circular DNA isolation and sequencing

To quantify eccDNAs during fly aging, 30 female flies were harvested from 5-days and 30-days post eclosion, and a fixed amount of pre-extraction plasmids was added prior to cell lysis: ~80 pg of ~7kb-pGL3-DmPiwipro1 and ~50 pg of ~11kb pJDS246-CarhCas9 (pJC9F3) About 30 ug of total gDNA was recovered from using MasterPure Complete DNA and RNA Purification kit (Lucigen), and 0.5ug-1ug gDNA was checked on a 1% agarose gel for integrity and quality. Good gDNA primarily migrated at >10kb and lack of RNA contamination that would result in bands at the lower molecular weight ranges. 20 μg gDNA were added to a 40ul of a second post-extraction plasmid cocktail: (1ng/ul of the 2.7kb pUC19, 0.1ng/ul of the 3.5kb pMaxGFP, 0.01ng/ul of the 5.2kb pGSH0 and 0.001ng/ul of the 6.3kb pCENPm3) and split equally to two reactions: Exo5/8 non-treated control versus Exo5/8 treated samples. We conducted a first round of Exo5/Exo8 (NEB) treatment at 37°C overnight, then an additional 2-hour treatment with freshly replenished buffer, ATP and enzymes. The reaction was stopped and purified using AMPure XP beads (Beckman Coulter) and eluted in 50 ul of water.

To check the efficiency of Exo5/8 treatment, 10 ul of the eluate from untreated versus treated samples were loaded on 1% agarose gel to visualize the completeness of digestion of gDNA. We quality controlled Exo5/8 treatments by performing qPCR against *Rp49*, ND5 and various plasmid primers including pUC19, pGL3piwipro and pCas9 and Ct values were compared between untreated versus treated samples. Mitochondria was not a reliable circular molecule because of the high variability of ND5 Ct values across multiple sample preps. Comparing between treated and untreated sample, the plasmids Ct values were generally stable (<2 Ct difference), and much higher for *Rp49* (>5 Ct difference) indicating the Exo5/8 treatments were effective at removing linear chromosomal DNA and not affecting the circular plasmids. Half of the Exo5/8 treated sample (25 ul out of 50 ul purified elute) was used as template for library construction using NEBNext UltraII library prep kit as stated above.

For eccDNA sequencing from brains, 200 female brains were dissected and added with half the volume of pre-extraction plasmids as whole flies, and gDNA concentration was measured by the Qubit 4 Fluorometer (Thermofisher). To 100 ng of brain gDNA, we mixed 20 ul of the post-extraction plasmids spike-in cocktail and a tenth of the Exo5/8 enzyme as whole flies gDNA. At least 10 million eccDNA reads were required for analysis. Libraries were pair-end sequenced at 36 bp by 36 bp on a Nextseq550 flow cell (Illumina).

### TIDAL updates with total TE consensus and gene mapping strategies

TE insertion analysis was carried out with an updated version of our previously developed TIDAL program (original code available on the Github repository at: https://github.com/laulabbrandeis/TIDAL) [65]. In this study, the updated version of TIDALv1.2 is also posted to Github at (https://github.com/laulabbumc/TIDAL1.2). These scripts carry out the analysis run the same way as the original TIDAL, but we incorporated two additional features. First, for the euchromatic TE insertions we selected 100 arbitrarily selected protein coding gene (Immobile gene elements IGEs) that are computed along with consensus TE sequence to benchmark noise in detection of genetic elements. The algorithm used to identify transposon insertion sites based on consensus transposon sequence is then applied on these 100 IGE sequence to determine their insertion sites. Second, for the total reads mapped to consensus TE sequences, here we added 100 IGEs are computed by mapping reads with bowtie2 using parameters “—sensitive—end-to-end” and custom shell, Perl, C-code, and R-code scripts all accessible from (https://github.com/laulabbumc/TIDAL1.2).

TEMP v1.05 code was acquired from the GitHub repository at: (https://github.com/JialiUMassWengLab/TEMP), and was run with default parameters except "-x 30, -m 3 -f 500". These parameters were chosen to ensure that TEMP results are consistent with analysis shown in [60,62].

### Downsampling and bootstrapping of TIDALv1.2 results

For each pair of 5d versus 30d samples, number of mapped reads were calculated by removing unmapped portion from the total reads (S1 Table) and the ratio of mapped reads between the lower depth versus deeper depth sample was calculated and round up to 4 significant digits. This ratio was used as the fraction number parameter by the Seqtk tool [119] for randomly selecting a fraction from the deeper depth library to the similar amount of the lower depth library. Mapped reads were computed and compared between each set of the libraries, leading to an average of an insignificant difference of less than 3000 reads.

For the initial bootstrapping of a testing *OreR* 30-day whole fly library for 100 permutations, Seqtk tool [119] was iterated 100 times with seed numbers set equal to the iteration number and fraction number set equal to 0.5. These 100 random downsampled fastq files were subsequently batch processed by TIDALv1.2 and TE insertion numbers for each permutation were counted. We plot the number of TE insertions per million of mapped reads for each permutation (S2A Fig) and calculated the standard deviation between each number of permutations run (increased number of permutations from 1 to 100, S2C Fig). We also plotted the cumulative average of TE insertions per million of mapped reads from increasing number of permutations (S2B Fig). Finally, we chose to set at 20 permutations for analyzing normalized TE insertions per million of mapped reads for all the libraries with error bar plotting as the 95% Confidence Interval.

After downsampling to equalize the library sizes between 5-day and 30-day samples, the differences in total number of TE insertion calls between the 30-day and 5-day samples were reassessed and recorded in S1 Table.

### Bioinformatics counting of eccDNA from TEs and spike-in plasmids using a custom pipeline and CIRCLE-Map program

In our first look at the eccDNA reads, we inputted them into an existing bioinformatics pipeline already developed for mapping *Drosophila* small RNA counts to TEs [120]. Reads were first checked by the Cutadapt program to see if adaptor sequences at the 3’ end needed to be removed, and then we indexed the reads to the *Drosophila* genome assembly file by running BWA version 1 [121] and formatdb from NCBI. Using Bowtie1 with 2 mismatches [122], reads were mapped to genome to get the genic and intergenic counts using the genome GTF file. The total number of reads mapped to the *Drosophila* genome was derived by subtracting the total number of reads not mapped to the *Drosophila* genome from the total number of reads. The total number of mapped reads was used as the basis for normalization of TE counts and spike-in plasmid counts.

Plasmid sequences were treated as linear entries in the FASTA file database similar to the TE family consensus sequences. The raw read counts from TE mapping were further normalized by the total number of reads mapping to the *Drosophila* Dm6 genome assembly. For spike-in plasmid counting, because several plasmids share the same backbone with different inserts, read frequencies were normalized by the total plasmid mapping sites as well as by the total number of *Drosophila* genome-mapping reads. Normalization using spike-ins was calculated as [(x/y)*10^6]^/z where x is the read counts, y is the total number of aligned reads from a given library and z is spike-in read counts.

To execute the CIRCLE-Map program for repeats [79], we indexed the *Drosophila* genome FASTA file by BWA. We then used the MEM algorithm under BWA to align reads against the *Drosophila* genome FASTA file. Next, we sorted the reads by alignment position within the resulting BAM file and indexed the resulting BAM file. Finally, we detected the circles by calling CIRCLE-Map program. The CIRCLE-Map program for repeats yields an output for reads with two high scoring alignments as these ones are indicative of circles formed from regions with homology.

For calculating the ratio of individual circular TE coverage between 5-day versus 30-day, the cumulative average of the mean base coverage from all types of circular DNA coordinates for each type of TE was calculated and the ratio was further normalized by the respective total reads after trimming (bottom panel, S3 Table). For normalization by spike-ins, the ratio was further normalized by the mean base coverage ratio of the pre-extraction plasmid pGL3-DmPiwipro1, which was consistently detected by the CIRCLE-Map program throughout all samples with top ranked circle scores.

## Supporting information

S1 Fig Variability in TE Landscapes (TLs) between TEMP and TIDAL programs applied to the same set of whole genome sequencing (WGS) libraries. (A) The WGS libraries from [63] study where TEMP was used to determine TE Landscapes has different results from the subsequent [60] study as well as being distinct from our re-analysis of the datasets in this study with TIDAL. (B) Fewer but more confident determinations of TE landscapes using TIDAL to reanalyze the WGS data originally analyzed by TEMP in [62]. (C) TIDAL re-analysis of the WGS data from [60], which only detects the TE insertion marked by the maroon dot because this is supported by pair end reads pairs and encompassing split reads shown in the example genome browser snapshots to the right. All the orange dots are likely false positives that TEMP will report but TIDAL is designed to filter. (D) Boxplot of TIDAL TE counts normalized to library depth of RPMs, and then the ratio of the normalized TE count between 21 day and 2–4 day flies from the *w1xHar* cross.
(PDF)

S2 Fig Bootstrapping analysis of TE insertion determinations by TIDAL from WGS data. (A) Using the *OreR* whole females WGS library data and downsampling to 50% depth, 100 permutations of randomly-downsampled WGS data were run through TIDAL and TE insertions at different Coverage Ratios (CR) as well as Immobile Genetic Elements (IGEs). (B) Cumulative average of TIDAL TE insertion determinations with increasing number of permutations. (C) Standard deviation of TIDAL TE insertions reach an optimally low minimum at 20 permutations in this *OreR* bootstrapping test. (D–G) Bar charts showing the average TIDAL TE insertion determinations with 95% confidence interval marked by the error bars for each determination after subjecting each of these libraries to a 50% downsampling and 20 permutations bootstrapping test to evaluate for potential sequencing sampling noise during TIDAL analysis. Each cohort corresponds to other analyses from Figs 2, 3 and 4. (H) Re-analysis of [60] WGS from fly brains with TIDAL and sequencing coverage analysis. Libraries grouped by triplicates with average TE insertions normalized by library depth and standard deviation plotted. One library each in young fly sample from the 250nt long read library is a major outlier causing the wide standard deviation. (I) Boxplots of the ratio of 30-day versus 5-day coverage of individual TE families (left) and 100 arbitrarily selected protein coding genes also used for IGE analysis (right) from concatenated libraries from all brain samples.
(PDF)

S3 Fig Control-FREEC plots of WGS coverage from fly strains used to measure TE profiles during fly aging. (A) Wild type fly lines libraries. (B) *Aubergine* and *AGO3* mutants libraries. (C) *Piwi* mutants libraries. (D) *AGO2* mutants libraries. (E) *Dcr2* mutants libraries.
(PDF)

S4 Fig Location-independent, sequencing coverage-based comparison of genomic TE loads during *Drosophila* aging. (A) Total TE RPM (top) and average TE (middle) read coverage for TE consensus families versus (bottom) the average read coverage for 100 arbitrarily selected protein-coding genes also used for IGE analysis were examined between 5-day and 30-day old female *Drosophila* wild-type strains. Wilcoxon rank-sum tests applied, with *p-*value<0.05 (*), <0.01 (**), <0.001 (***). (B) Boxplot distributions of the ratios of sequencing coverage between 5-day and 30-day old flies for individual TE families (top), protein-coding genes (middle) and normalized TE coverage by gene coverage (bottom). (C) Individual TEs per genome quantified by droplet digital PCR in *ISO1* (top), *OreR* (middle) and *w1118* (bottom) strains from a standard input gDNA from 5-day versus 30-day old whole females. Errors bars are the propagated 95%confidence intervals from the absolute quantitation based upon the Poisson distribution. (D) Boxplot of the data in (C) confirms the lack of statistical significance in TE copy number difference between young and old WT flies.
(PDF)

S5 Fig Supporting WGS/TIDAL TE insertion counts with droplet digital PCR (ddPCR). (A) *P-elements* copies amongst high and low copy strains determined by WGS/TIDAL in Srivastav et al, 2019 are validated by droplet digital PCR. (B) *I-element* copies from *w1118* and *ISO1* from WGS/TIDAL are also validated by droplet digital PCR. (C) Genetic scheme for repeating the natural bursts of *I-element* transposition during oogenesis, derived from Wang et al, 2018. (D) The qPCR showing the fold higher copy number of *I-element* in the active parental strain versus the control strain using two concentrations of input DNA. (E) ddPCR absolute quantification of *I-element* copies per haploid genome from P0 females, F1 females, and F2 oocytes of Active *I-element* versus control strains. All ddPCR error bars represent the propagated 95% confidence interval of the Poisson distribution used by the ddPCR quantitation algorithm.
(PDF)

S6 Fig Visualizing enrichment of TEs, satellite repeats, and mitochondrial DNA from eccDNA-seq. Genome browser plots for (A) *copia*, (B) *gypsy*, (C) a Chr3R satellite repeat, and (D) *Drosophila* mitochondrial DNA loci with plots of WGS from gDNA on top in black, in the middle are red tracks of eccDNA sequences as RPM counts not normalized to plasmid spike in, and on the bottom are the Repeatmasker tracks. (E) EccDNAs with significant “circle scores” from the brains of *OreR* and *w1118* show increases during aging. (F) Ratio of 30-day/5-day reads from eccDNA libraries mapped to the list of 100 IGEs in the TIDAL v1.2 pipeline. (G) Box plots of 30-day/5-day ratios of read coverage for eccDNA TE sequences rated by our own custom quantitation pipeline that uses a TE-mapping scripts previously used for small RNA analysis. (H) Dot graph highlighting specific TE eccDNAs whose 30-day/5-day sequencing ratios are normalized to the RPM library size (open) or further normalized to the plasmid spike-ins (closed) from (G). (I) Boxplot showing an earlier Round-9 eccDNA library contained too much of the plasmid spike-ins to be useful for TE eccDNA analysis, compared to the subsequent Round-10 eccDNA library where plasmid spike-in reads are comparable to levels of TE eccDNA reads. (J) Relatively even levels of the individual plasmid spike-ins between samples evaluated against a fifth plasmid that was added to each set of flies before gDNA extraction, indicating that pipetting differences are not largely distorting the eccDNA quantifications.
(PDF)

S7 Fig Additional assessment of TE landscapes in other RNAi mutants. Quantification of new TE insertions as compared to the reference genome at different Coverage Ratios (CR) using the TIDAL-fly program from whole flies (A) and brains (B) of two *dicer-2* (*Dcr-2*) mutants. Additional TE insertion quantifications of (C) *AGO3* null mutants and (D) transgenic rescue of the *AGO2* gene into the *AGO2 (2-5-14)* null mutant. Asterisks mark the library that was down-sampled to the equivalent depth of the cognate comparison library.
(PDF)

S8 Fig Trials with brain-specific RNAi knockdown of *PAF1* with *elav-GAL4*. (A) Expressed as early as embryos, the *Elav-Gal4* driving *PAF1* knockdown in brains of adult *Drosophila* causes TE reduction in left graph without affecting control genes in the right graph. However, the early start of *elav-Gal4-PAF1-RNAi* also caused lifespan reduction in lifespan curve below (*p*<0.001) (B) Genomic PCR (top) and functional confirmation of the recombinant *elav-Gal4*,*Gal80*^*ts*^ stock (bottom). Crosses to include *Gal80*^*ts*^ with *elav-Gal4* enabled later adult as well as third-instar larvae induction as visualized by neuronal GFP expression. (C) Despite now using heat shock of *elav-Gal4*,*Gal80*^*ts*^ to drive neuron-specific *PAF1* RNAi, the knockdown was ineffective at 5-days post-eclosion (top graphs) with just some knockdown at 30-days post eclosion (bottom graph). (D) As a result, there is no significant lifespan extension with the *elav-Gal4*,*Gal80*^*ts*^ system. Control RNAi [*mCherry-shRNA/+; elavGa4*,*tubGal80*^*ts*^*/+*] and *PAF1 RNAi* [*elavGal4*,*tubGal80*^*ts*^*/PAF1RNAi]* females were induced at 29°C since day 1 adult. Both Ctrl and *PAF1* RNAi replicates are identical in genotype except replicate 2 are both heterozygous for the TM6 balancer on Chr3. (E) Additional knockdown of *PAF1* using the *Geneswitch ->elav-Gal4* system from third instar larvae with the drug RU486 versus ethanol carrier showed broader overall gene knockdown, and this resulted in a slightly protracted life-span extension. Chisq = 4.1 on 1 degrees of freedom, *p*<0.05.
(PDF)

S1 Table Statistics of the rounds of whole genome sequencing (WGS) using TIDALv1.2. (PDF)

S2 Table Oligonucleotide primers used in this study. (PDF)

S3 Table Statistics for the eccDNA sequencing. (PDF)
