# Supplementary material for: Transposable element landscapes in aging Drosophila
Source: PLoS Genet. 2022 Mar 3;18(3):e1010024. doi: 10.1371/journal.pgen.1010024 (PMC8893327; doi:10.1371/journal.pgen.1010024)
Supplement: S1 Fig — (A) The WGS libraries from [63] study where TEMP was used to determine TE Landscapes has different results from the subsequent [60] study as well as being distinct from our re-analysis of the datasets in this study with TIDAL. (B) Fewer but more confident determinations of TE landscapes using TIDAL to reanalyze the WGS data originally analyzed by TEMP in [62]. (C) TIDAL re-analysis of the WGS data from [60], which only detects the TE insertion marked by the maroon dot because this is supported by pair end reads pairs and encompassing split reads shown in the example genome browser snapshots to the right. All the orange dots are likely false positives that TEMP will report but TIDAL is designed to filter. (D) Boxplot of TIDAL TE counts normalized to library depth of RPMs, and then the ratio of the normalized TE count between 21 day and 2–4 day flies from the w1xHar cross. (PDF) [file pgen.1010024.s001.pdf]

Yang, et al.
S1 Fig.

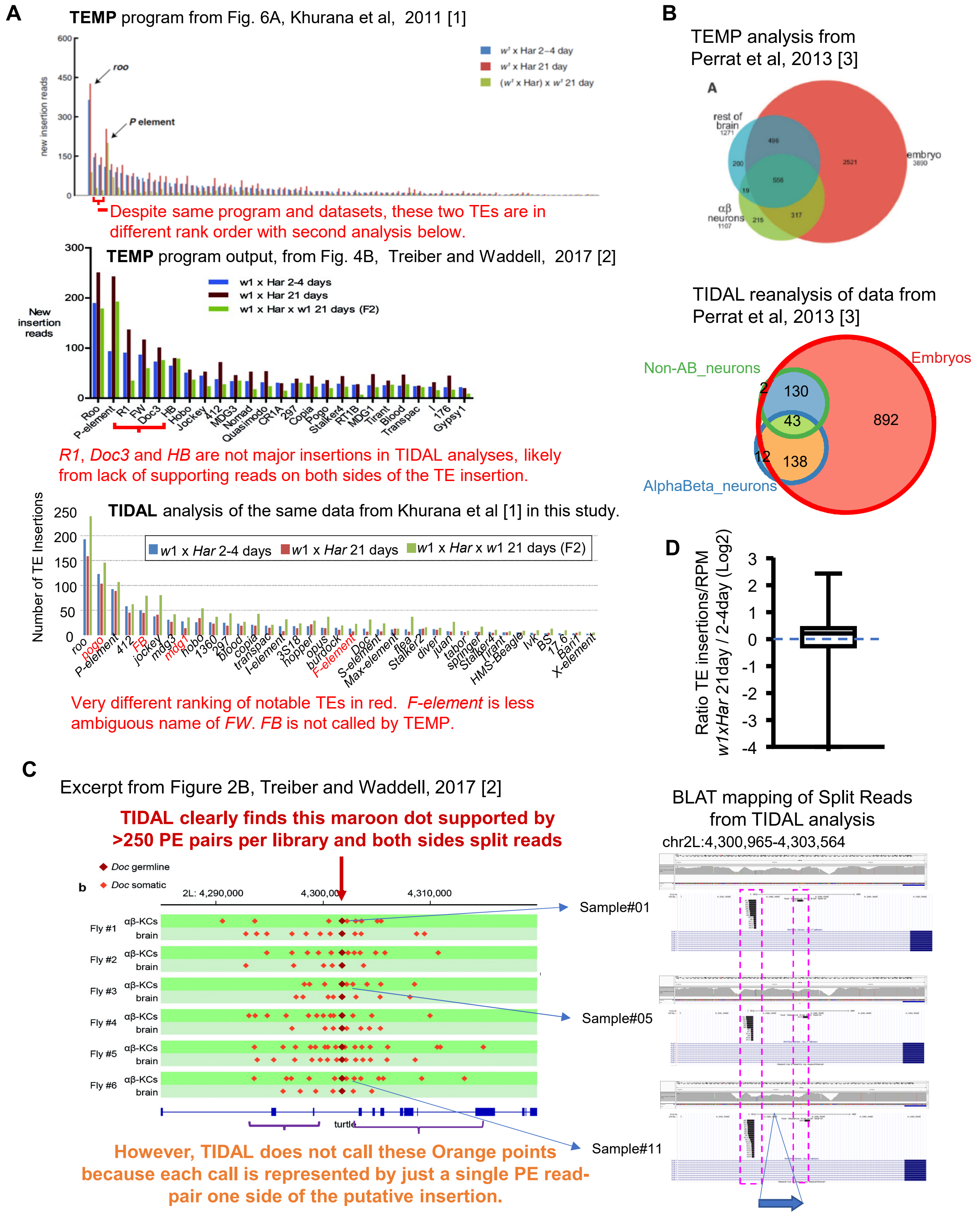

1.Khurana JS, Wang J, Xu J, Koppetsch BS, Thomson TC, Nowosielska A, Li C, Zamore PD, Weng Z, Theurkauf WE. 2011. Adaptation to P element transposon invasion in Drosophila melanogaster. *Cell* **147**: 1551-1563.
2. Treiber CD, Waddell S. 2017. Resolving the prevalence of somatic transposition in Drosophila. *eLife* **6**.
3. Perrat PN, DasGupta S, Wang J, Theurkauf W, Weng Z, Rosbash M, Waddell S. 2013. Transposition-driven genomic heterogeneity in the Drosophila brain. *Science* **340**: 91-95.
