# Supplementary figures and images for: Transposable element landscapes in aging Drosophila
Source: PLoS Genet. 2022 Mar 3;18(3):e1010024. doi: 10.1371/journal.pgen.1010024 (PMC8893327; doi:10.1371/journal.pgen.1010024)

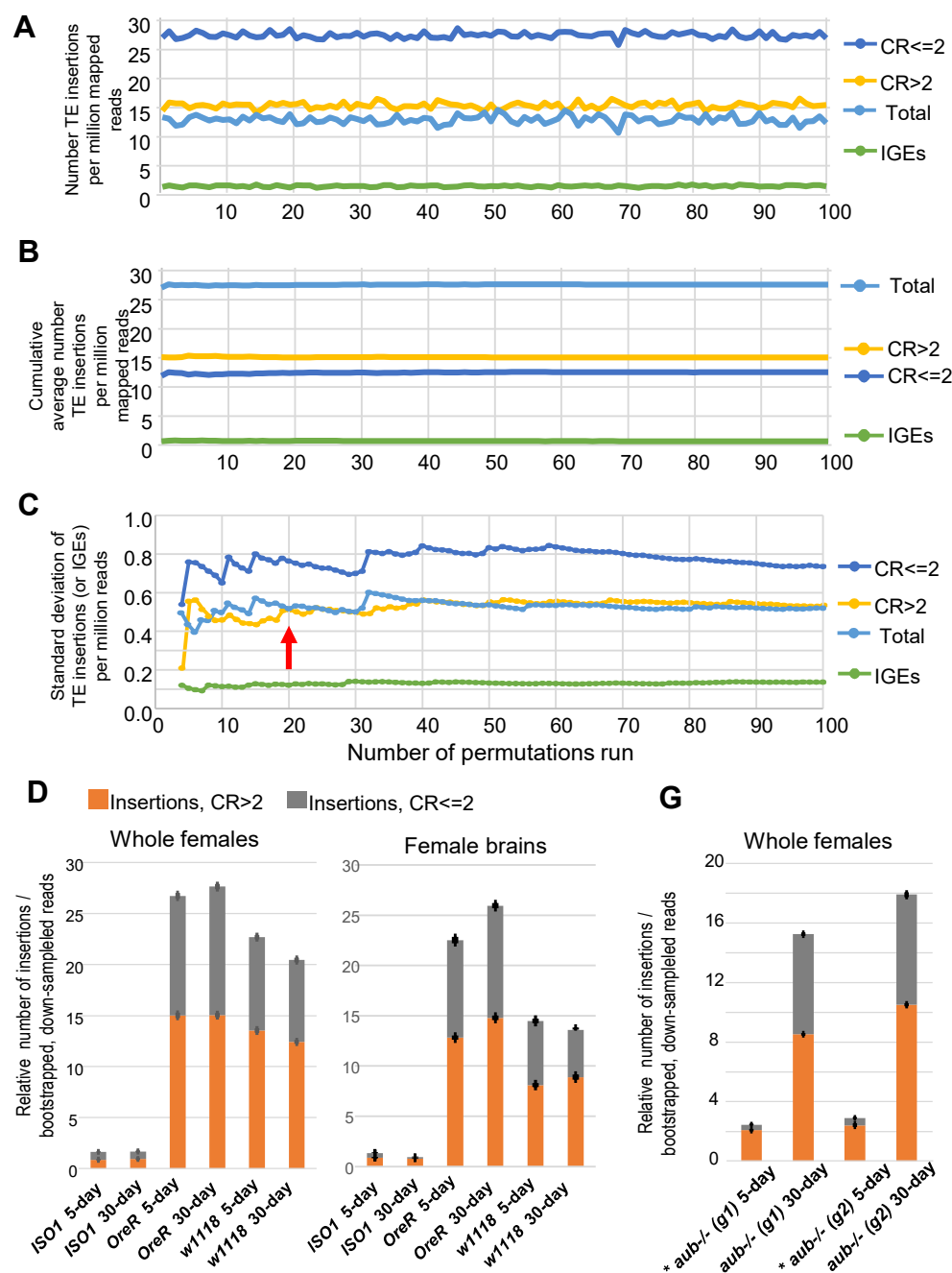

Supplement: S2 Fig — (A) Using the OreR whole females WGS library data and downsampling to 50% depth, 100 permutations of randomly-downsampled WGS data were run through TIDAL and TE insertions at different Coverage Ratios (CR) as well as Immobile Genetic Elements (IGEs). (B) Cumulative average of TIDAL TE insertion determinations with increasing number of permutations. (C) Standard deviation of TIDAL TE insertions reach an optimally low minimum at 20 permutations in this OreR bootstrapping test. (D–G) Bar charts showing the average TIDAL TE insertion determinations with 95% confidence interval marked by the error bars for each determination after subjecting each of these libraries to a 50% downsampling and 20 permutations bootstrapping test to evaluate for potential sequencing sampling noise during TIDAL analysis. Each cohort corresponds to other analyses from Figs 2, 3 and 4. (H) Re-analysis of [60] WGS from fly brains with TIDAL and sequencing coverage analysis. Libraries grouped by triplicates with average TE insertions normalized by library depth and standard deviation plotted. One library each in young fly sample from the 250nt long read library is a major outlier causing the wide standard deviation. (I) Boxplots of the ratio of 30-day versus 5-day coverage of individual TE families (left) and 100 arbitrarily selected protein coding genes also used for IGE analysis (right) from concatenated libraries from all brain samples. (PDF) [file pgen.1010024.s002.pdf]

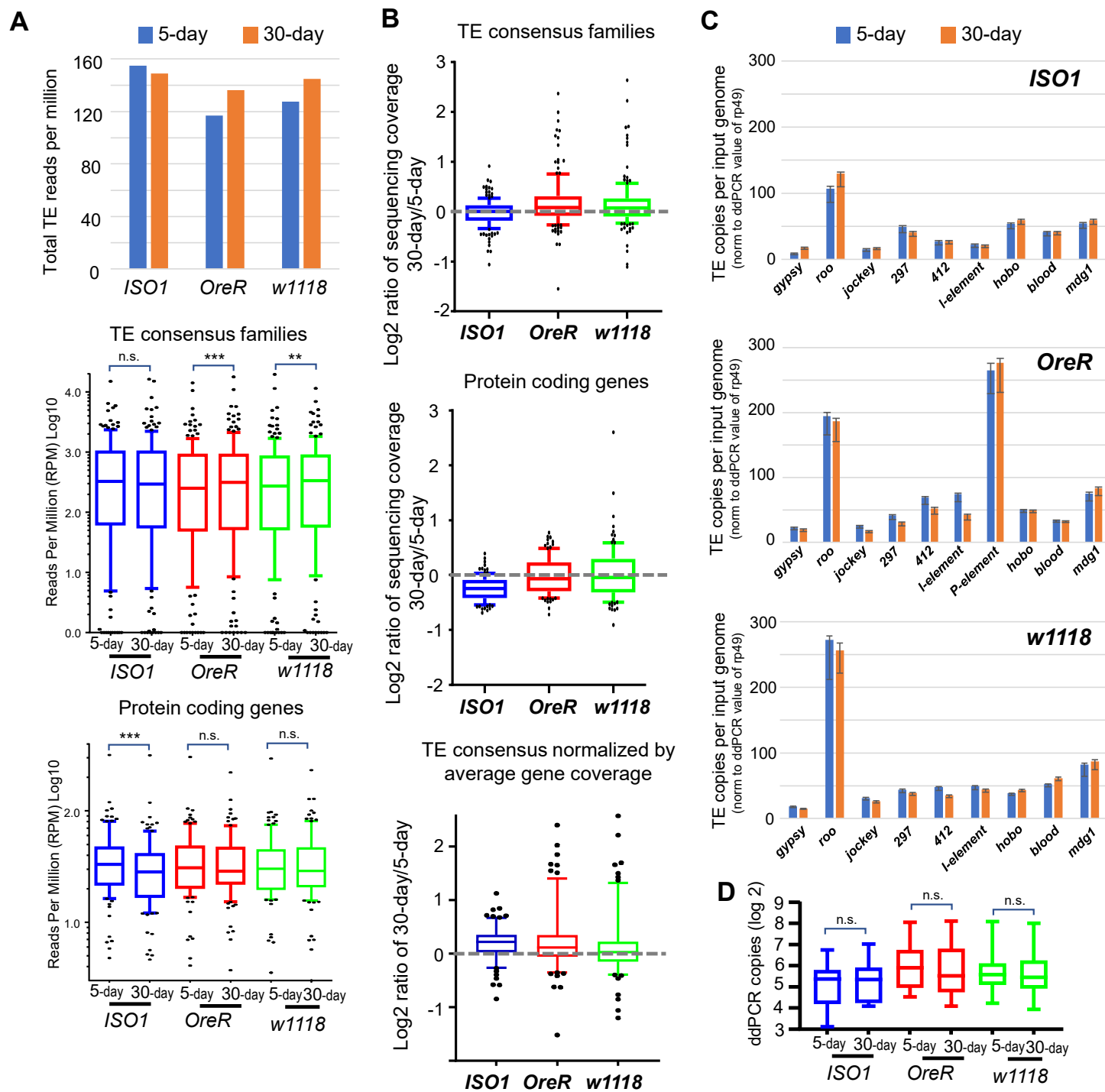

Supplement: S4 Fig — (A) Total TE RPM (top) and average TE (middle) read coverage for TE consensus families versus (bottom) the average read coverage for 100 arbitrarily selected protein-coding genes also used for IGE analysis were examined between 5-day and 30-day old female Drosophila wild-type strains. Wilcoxon rank-sum tests applied, with p-value<0.05 (*), <0.01 (**), <0.001 (***). (B) Boxplot distributions of the ratios of sequencing coverage between 5-day and 30-day old flies for individual TE families (top), protein-coding genes (middle) and normalized TE coverage by gene coverage (bottom). (C) Individual TEs per genome quantified by droplet digital PCR in ISO1 (top), OreR (middle) and w1118 (bottom) strains from a standard input gDNA from 5-day versus 30-day old whole females. Errors bars are the propagated 95%confidence intervals from the absolute quantitation based upon the Poisson distribution. (D) Boxplot of the data in (C) confirms the lack of statistical significance in TE copy number difference between young and old WT flies. (PDF) [file pgen.1010024.s004.pdf]

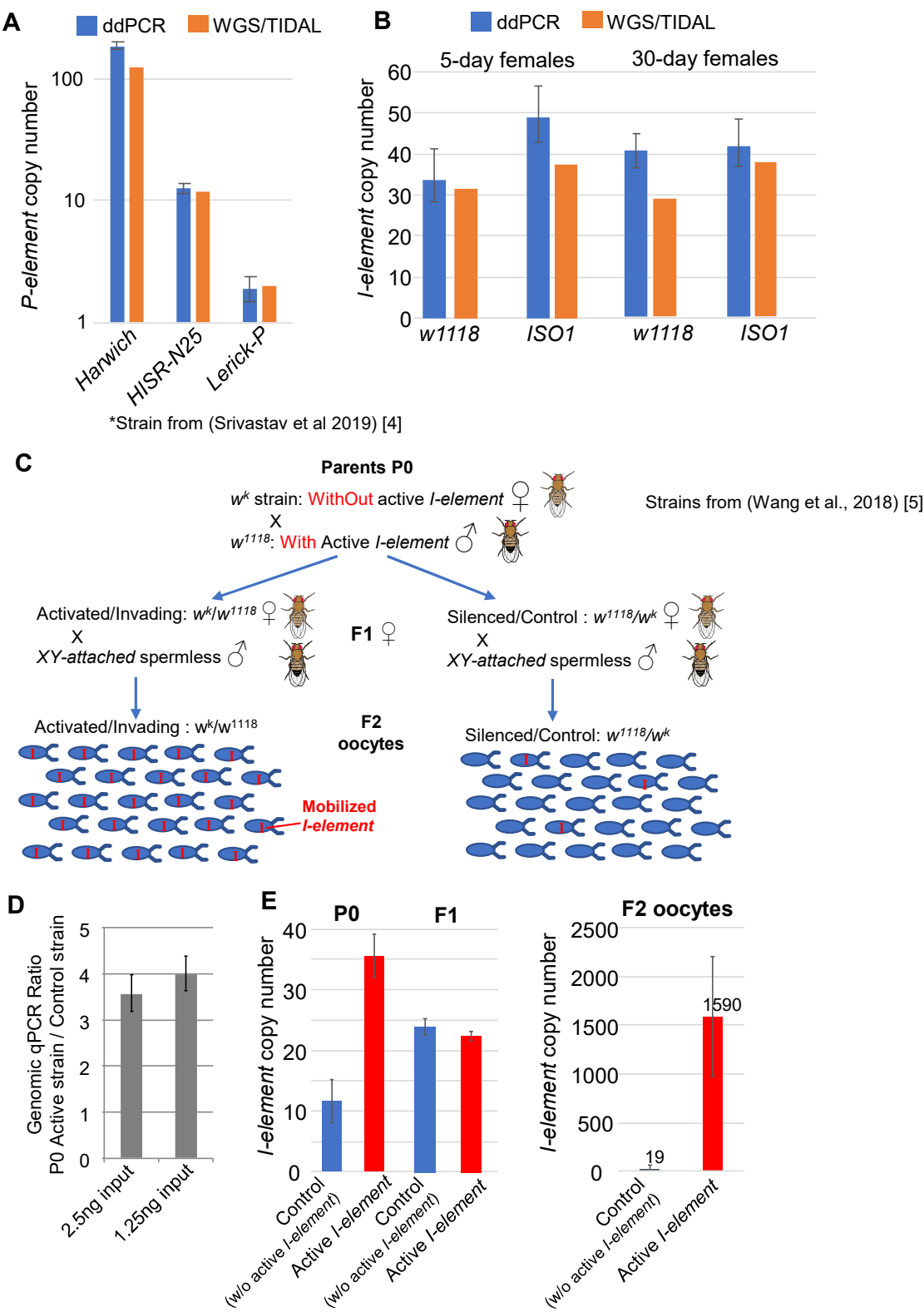

Supplement: S5 Fig — (A) P-elements copies amongst high and low copy strains determined by WGS/TIDAL in Srivastav et al, 2019 are validated by droplet digital PCR. (B) I-element copies from w1118 and ISO1 from WGS/TIDAL are also validated by droplet digital PCR. (C) Genetic scheme for repeating the natural bursts of I-element transposition during oogenesis, derived from Wang et al, 2018. (D) The qPCR showing the fold higher copy number of I-element in the active parental strain versus the control strain using two concentrations of input DNA. (E) ddPCR absolute quantification of I-element copies per haploid genome from P0 females, F1 females, and F2 oocytes of Active I-element versus control strains. All ddPCR error bars represent the propagated 95% confidence interval of the Poisson distribution used by the ddPCR quantitation algorithm. (PDF) [file pgen.1010024.s005.pdf]

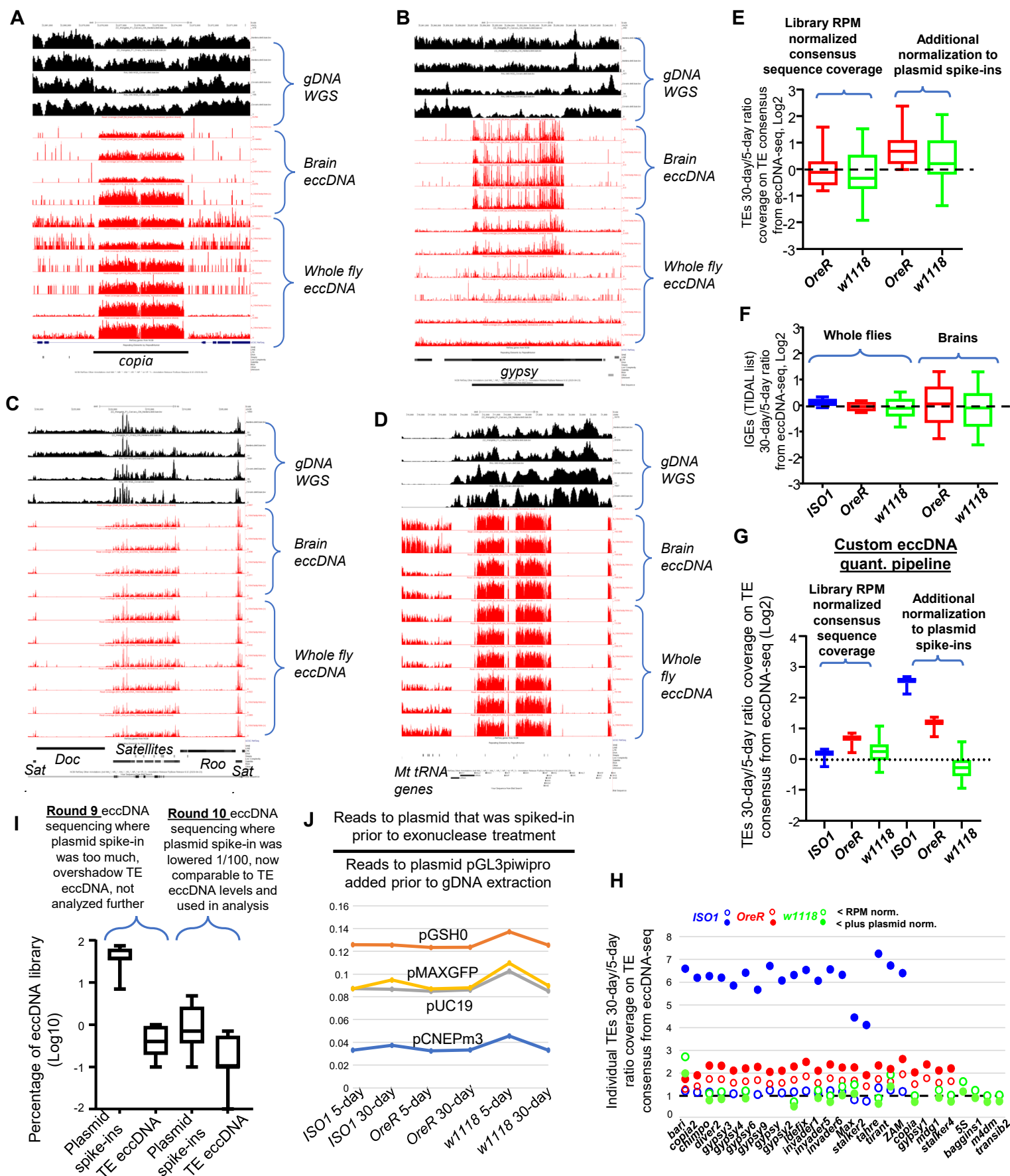

Supplement: S6 Fig — Genome browser plots for (A) copia, (B) gypsy, (C) a Chr3R satellite repeat, and (D) Drosophila mitochondrial DNA loci with plots of WGS from gDNA on top in black, in the middle are red tracks of eccDNA sequences as RPM counts not normalized to plasmid spike in, and on the bottom are the Repeatmasker tracks. (E) EccDNAs with significant “circle scores” from the brains of OreR and w1118 show increases during aging. (F) Ratio of 30-day/5-day reads from eccDNA libraries mapped to the list of 100 IGEs in the TIDAL v1.2 pipeline. (G) Box plots of 30-day/5-day ratios of read coverage for eccDNA TE sequences rated by our own custom quantitation pipeline that uses a TE-mapping scripts previously used for small RNA analysis. (H) Dot graph highlighting specific TE eccDNAs whose 30-day/5-day sequencing ratios are normalized to the RPM library size (open) or further normalized to the plasmid spike-ins (closed) from (G). (I) Boxplot showing an earlier Round-9 eccDNA library contained too much of the plasmid spike-ins to be useful for TE eccDNA analysis, compared to the subsequent Round-10 eccDNA library where plasmid spike-in reads are comparable to levels of TE eccDNA reads. (J) Relatively even levels of the individual plasmid spike-ins between samples evaluated against a fifth plasmid that was added to each set of flies before gDNA extraction, indicating that pipetting differences are not largely distorting the eccDNA quantifications. (PDF) [file pgen.1010024.s006.pdf]

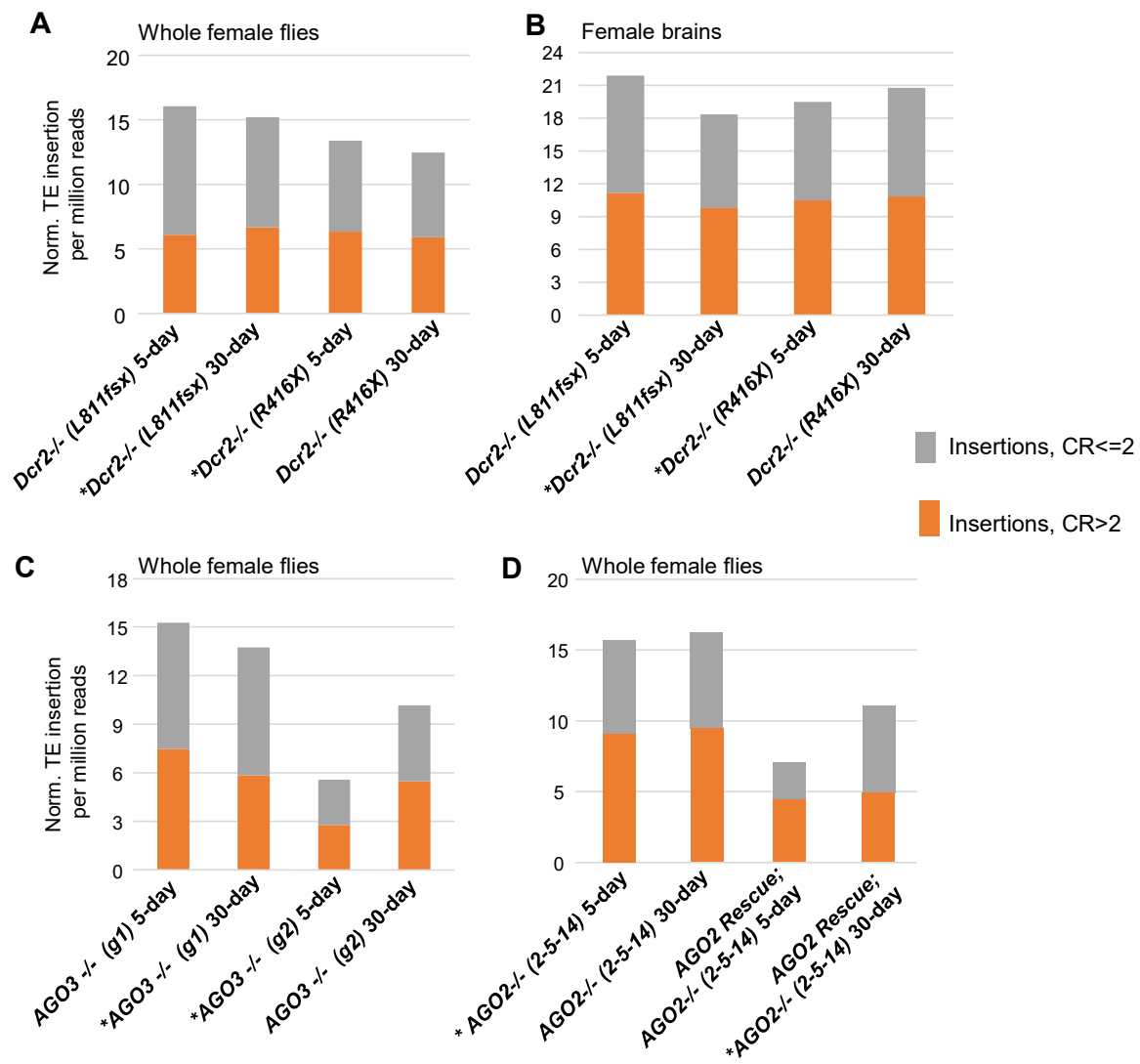

Supplement: S7 Fig — Quantification of new TE insertions as compared to the reference genome at different Coverage Ratios (CR) using the TIDAL-fly program from whole flies (A) and brains (B) of two dicer-2 (Dcr-2) mutants. Additional TE insertion quantifications of (C) AGO3 null mutants and (D) transgenic rescue of the AGO2 gene into the AGO2 (2-5-14) null mutant. Asterisks mark the library that was down-sampled to the equivalent depth of the cognate comparison library. (PDF) [file pgen.1010024.s007.pdf]

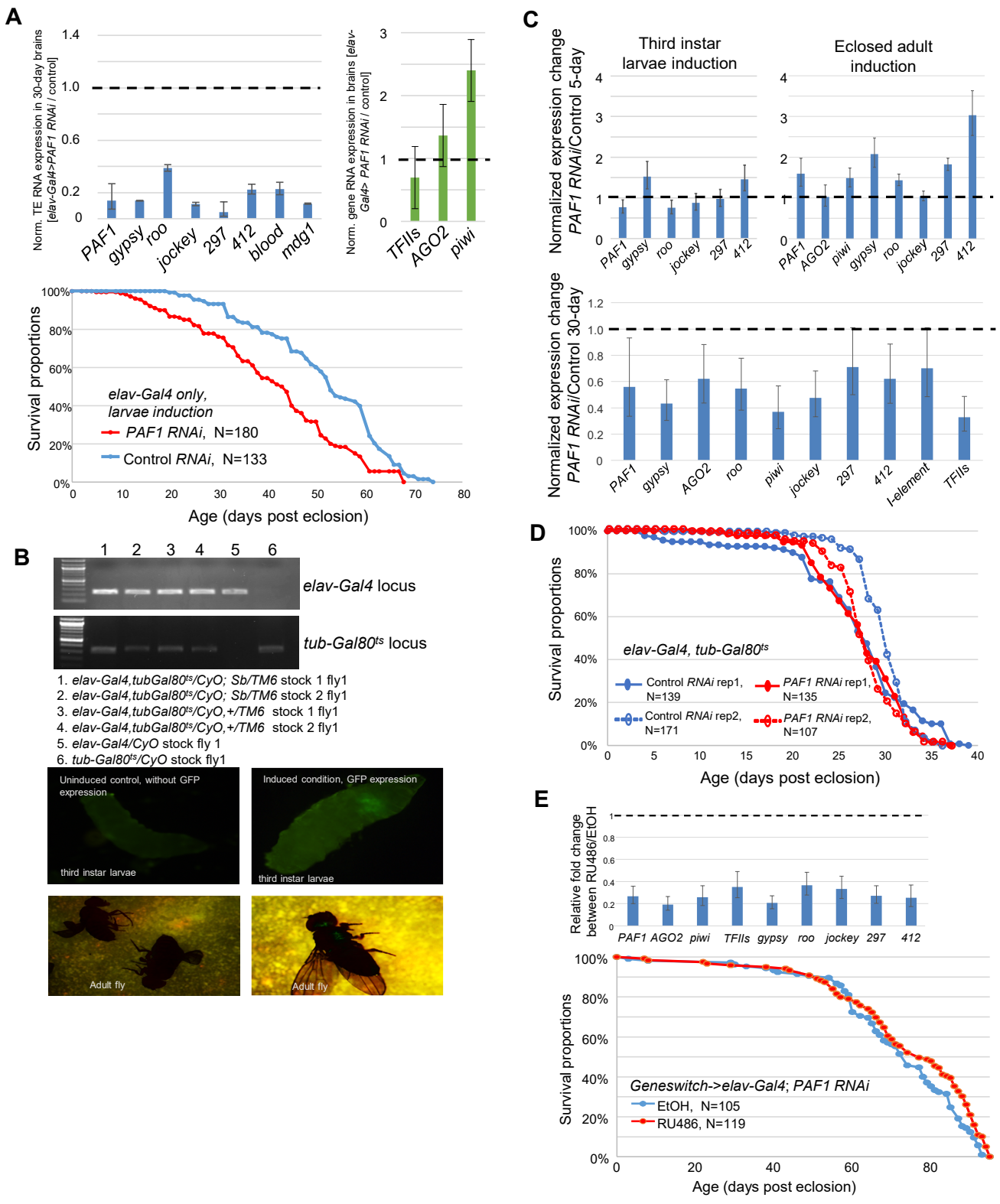

Supplement: S8 Fig — (A) Expressed as early as embryos, the Elav-Gal4 driving PAF1 knockdown in brains of adult Drosophila causes TE reduction in left graph without affecting control genes in the right graph. However, the early start of elav-Gal4-PAF1-RNAi also caused lifespan reduction in lifespan curve below (p<0.001) (B) Genomic PCR (top) and functional confirmation of the recombinant elav-Gal4,Gal80ts stock (bottom). Crosses to include Gal80ts with elav-Gal4 enabled later adult as well as third-instar larvae induction as visualized by neuronal GFP expression. (C) Despite now using heat shock of elav-Gal4,Gal80ts to drive neuron-specific PAF1 RNAi, the knockdown was ineffective at 5-days post-eclosion (top graphs) with just some knockdown at 30-days post eclosion (bottom graph). (D) As a result, there is no significant lifespan extension with the elav-Gal4,Gal80ts system. Control RNAi [mCherry-shRNA/+; elavGa4,tubGal80ts/+] and PAF1 RNAi [elavGal4,tubGal80ts/PAF1RNAi] females were induced at 29°C since day 1 adult. Both Ctrl and PAF1 RNAi replicates are identical in genotype except replicate 2 are both heterozygous for the TM6 balancer on Chr3. (E) Additional knockdown of PAF1 using the Geneswitch ->elav-Gal4 system from third instar larvae with the drug RU486 versus ethanol carrier showed broader overall gene knockdown, and this resulted in a slightly protracted life-span extension. Chisq = 4.1 on 1 degrees of freedom, p<0.05. (PDF) [file pgen.1010024.s008.pdf]
