# Supplementary material for: Transposable element landscapes in aging Drosophila
Source: PLoS Genet. 2022 Mar 3;18(3):e1010024. doi: 10.1371/journal.pgen.1010024 (PMC8893327; doi:10.1371/journal.pgen.1010024)

Yang, et al.
S3 Fig.

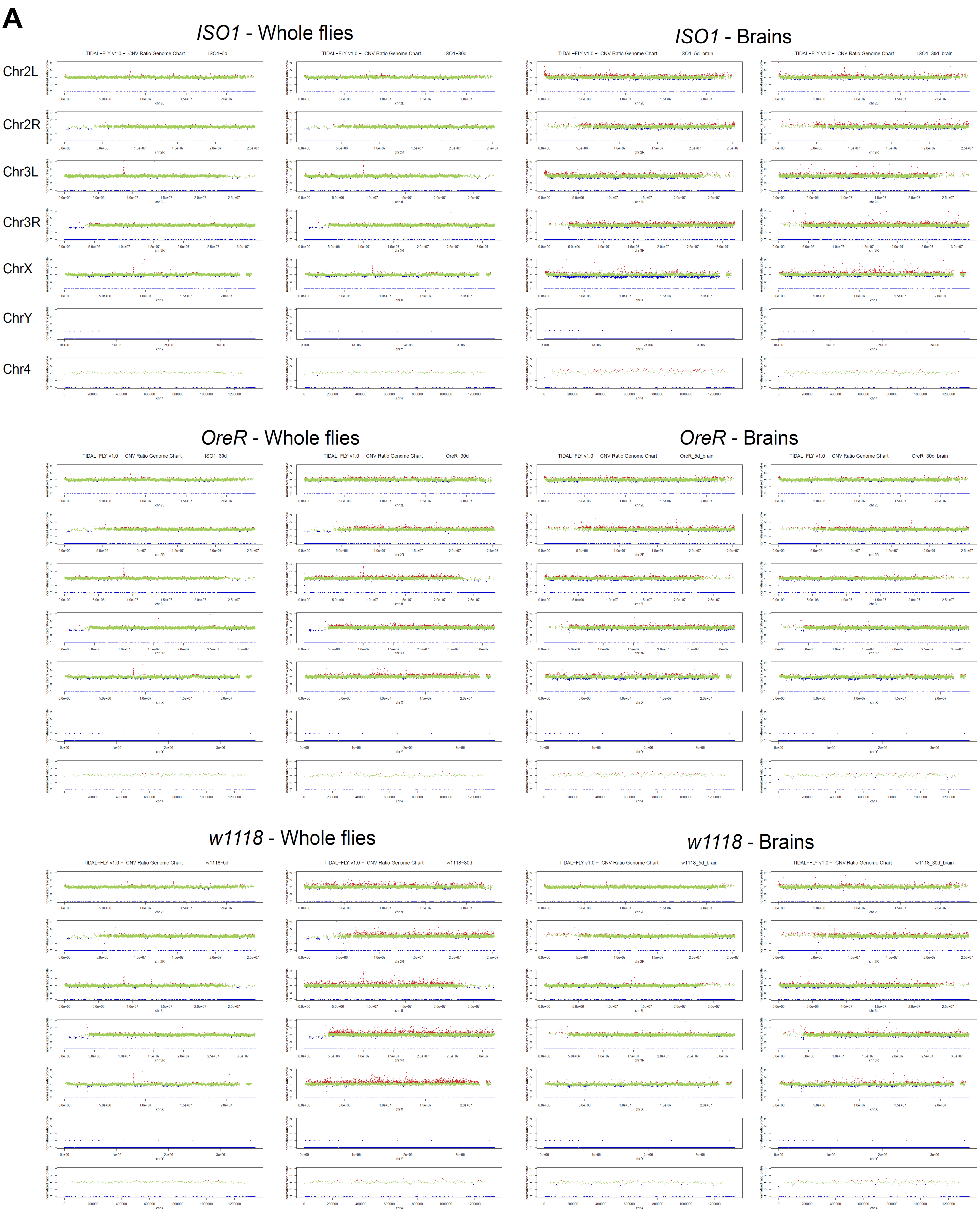

Yang, et al.
S3 Fig.

B

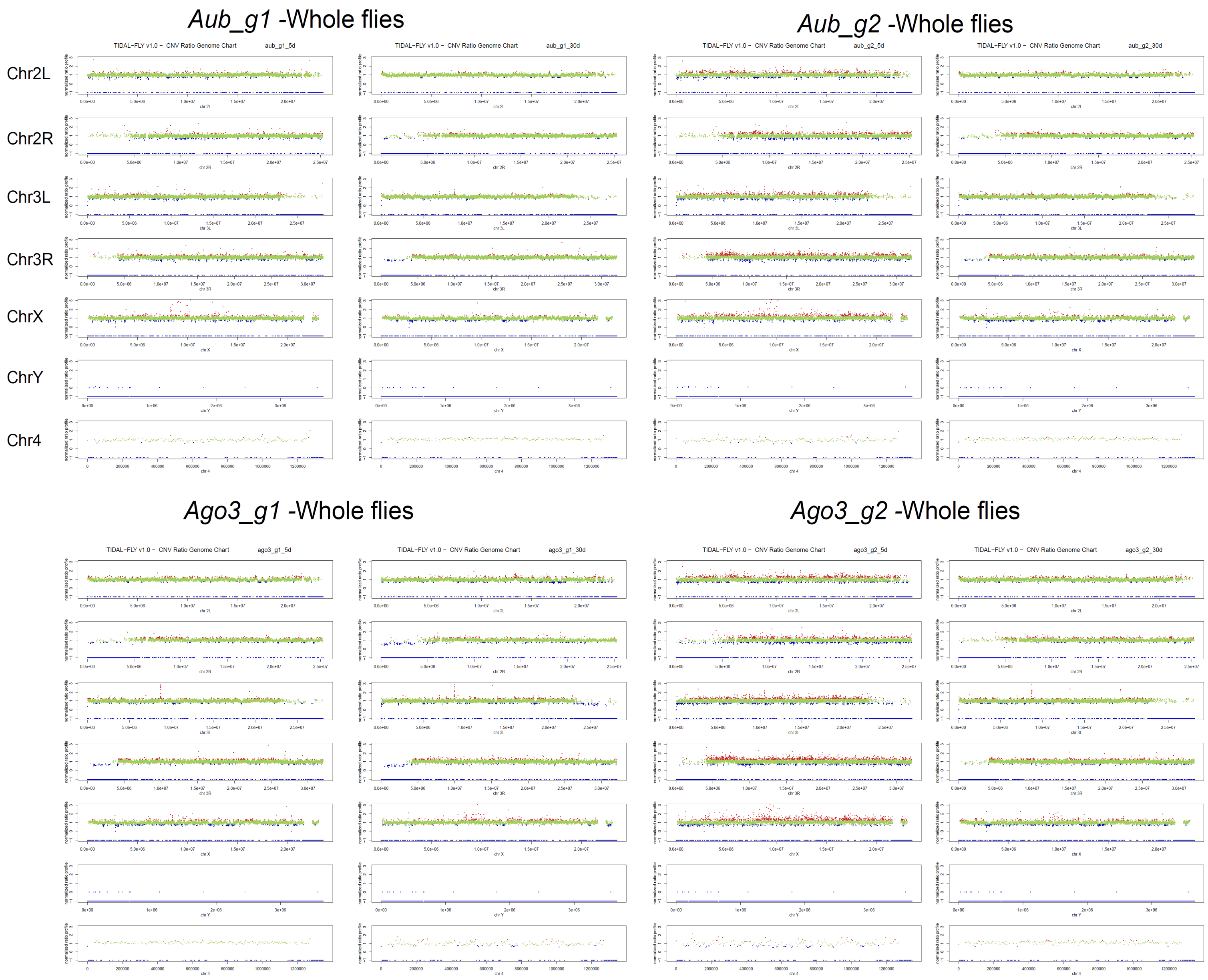

Yang, et al.
S3 Fig.

C

PIWI_g1 -Whole flies

PIWI_g1 -Brains

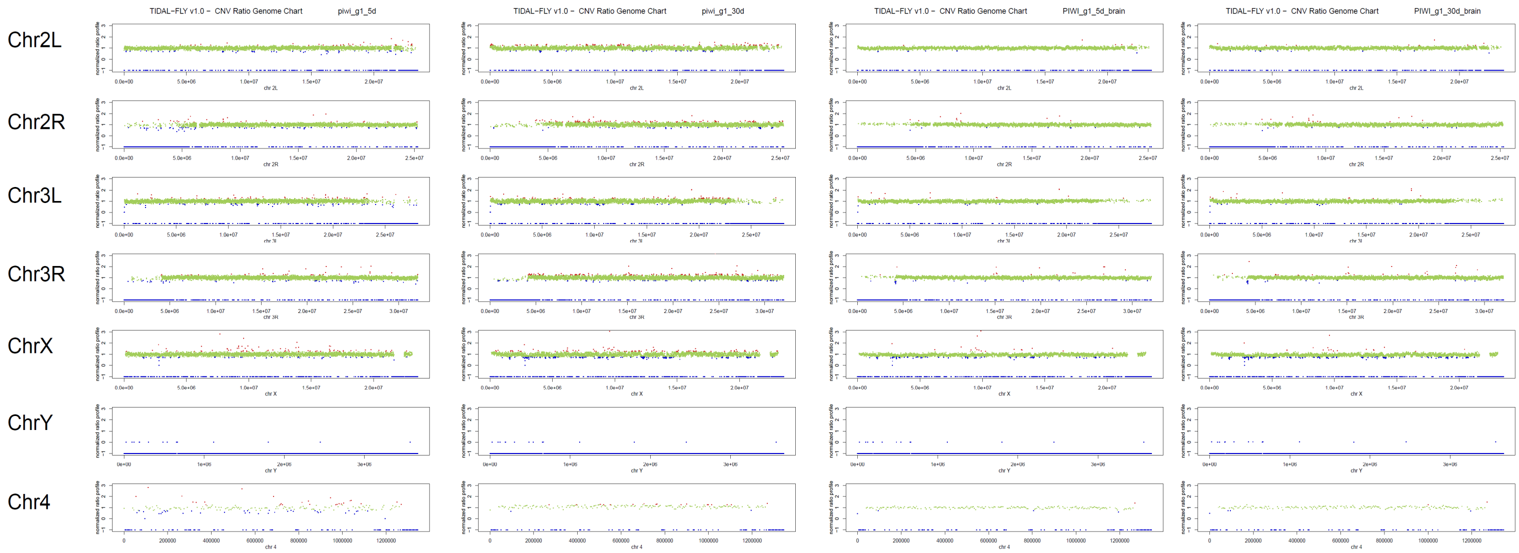

PIWI[HDR-4xP3-mcherry] -Whole flies

PIWI[HDR-4xP3-mcherry] -Brains

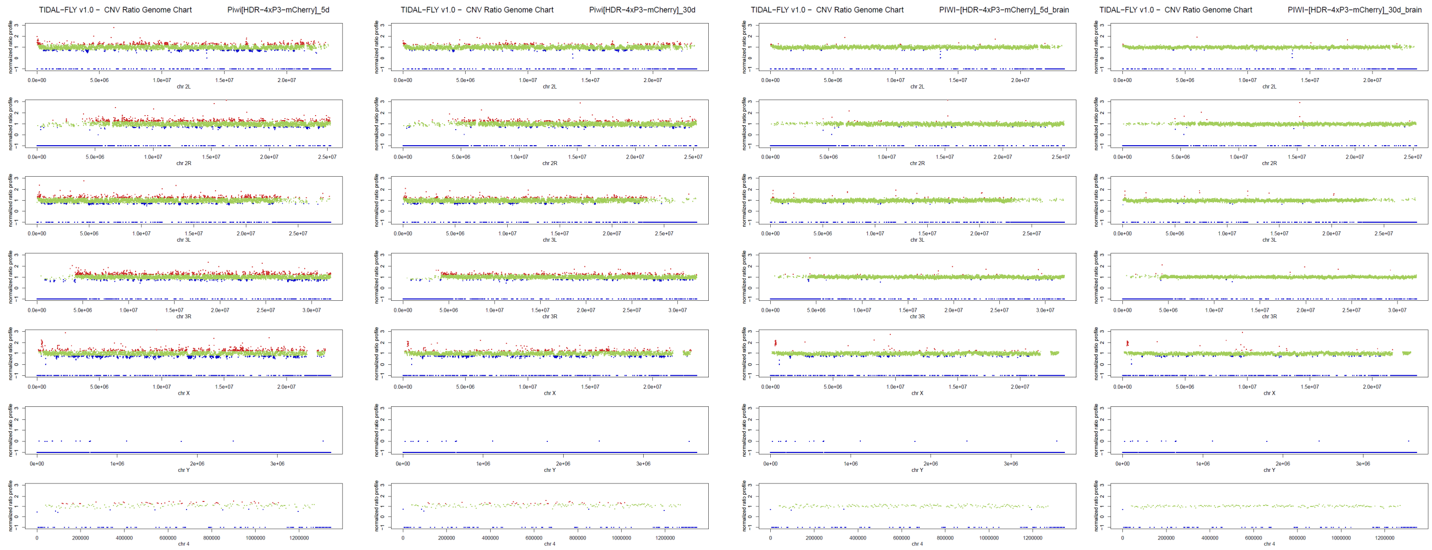

D

Ago2_2-5-14 -Whole flies   *Ago2_2-5-14* -Brains

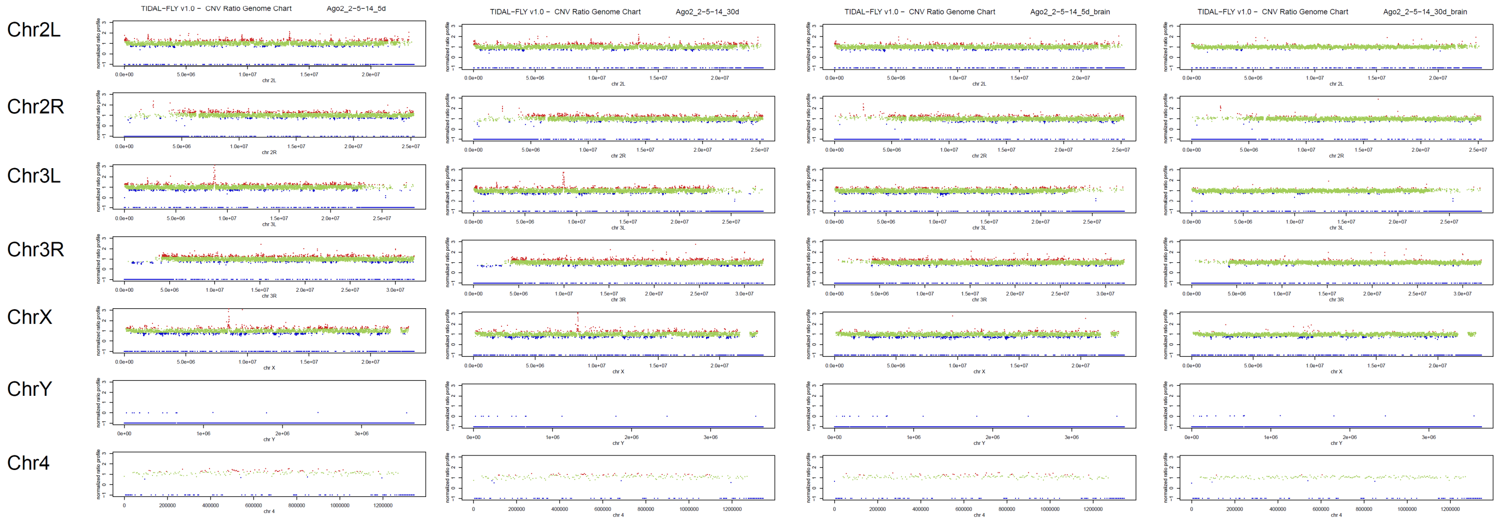

Ago2_2-16-4 -Whole flies   *Ago2_2-16_4* -Brains

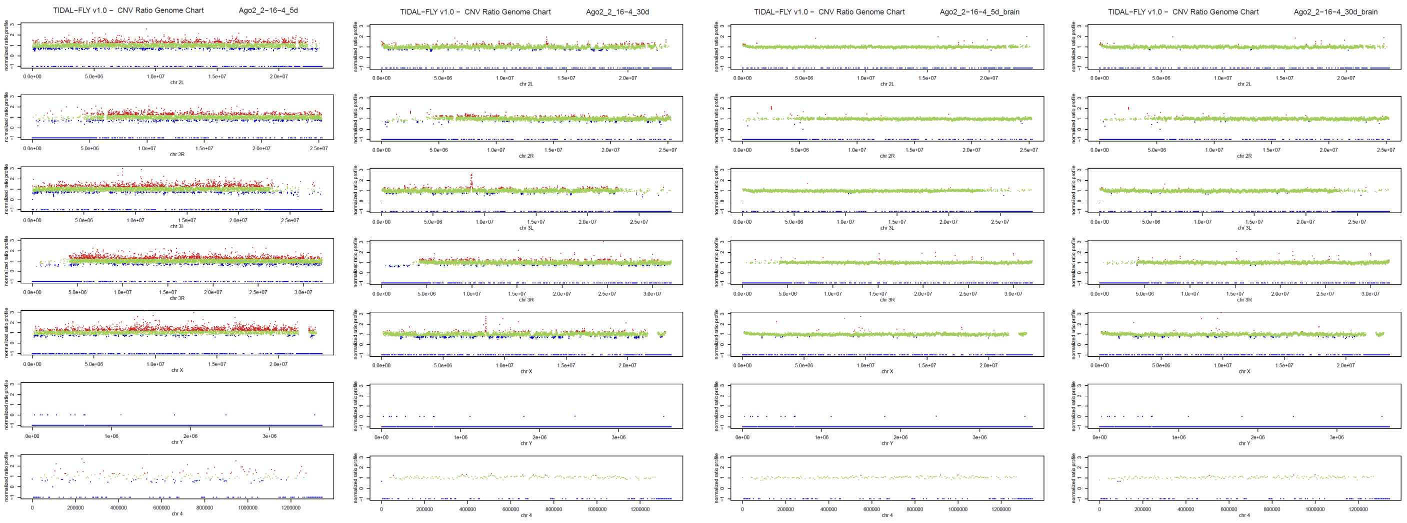

WT-Ago2 _Rescue_Ago2-2-5-14 -Whole flies

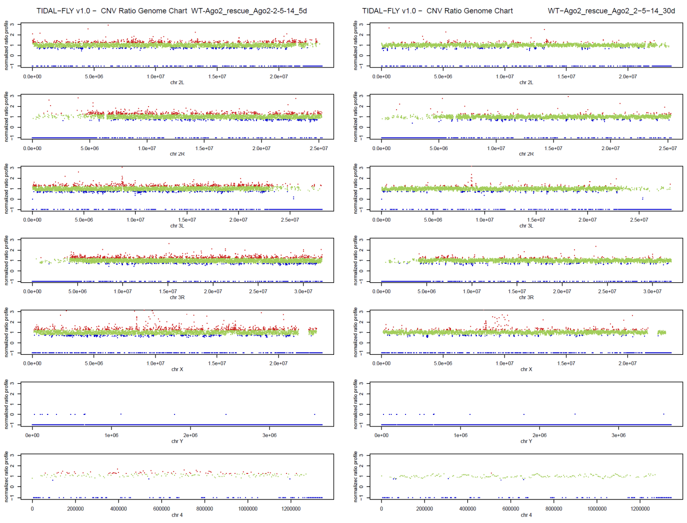

Yang, et al.
S3 Fig.

E

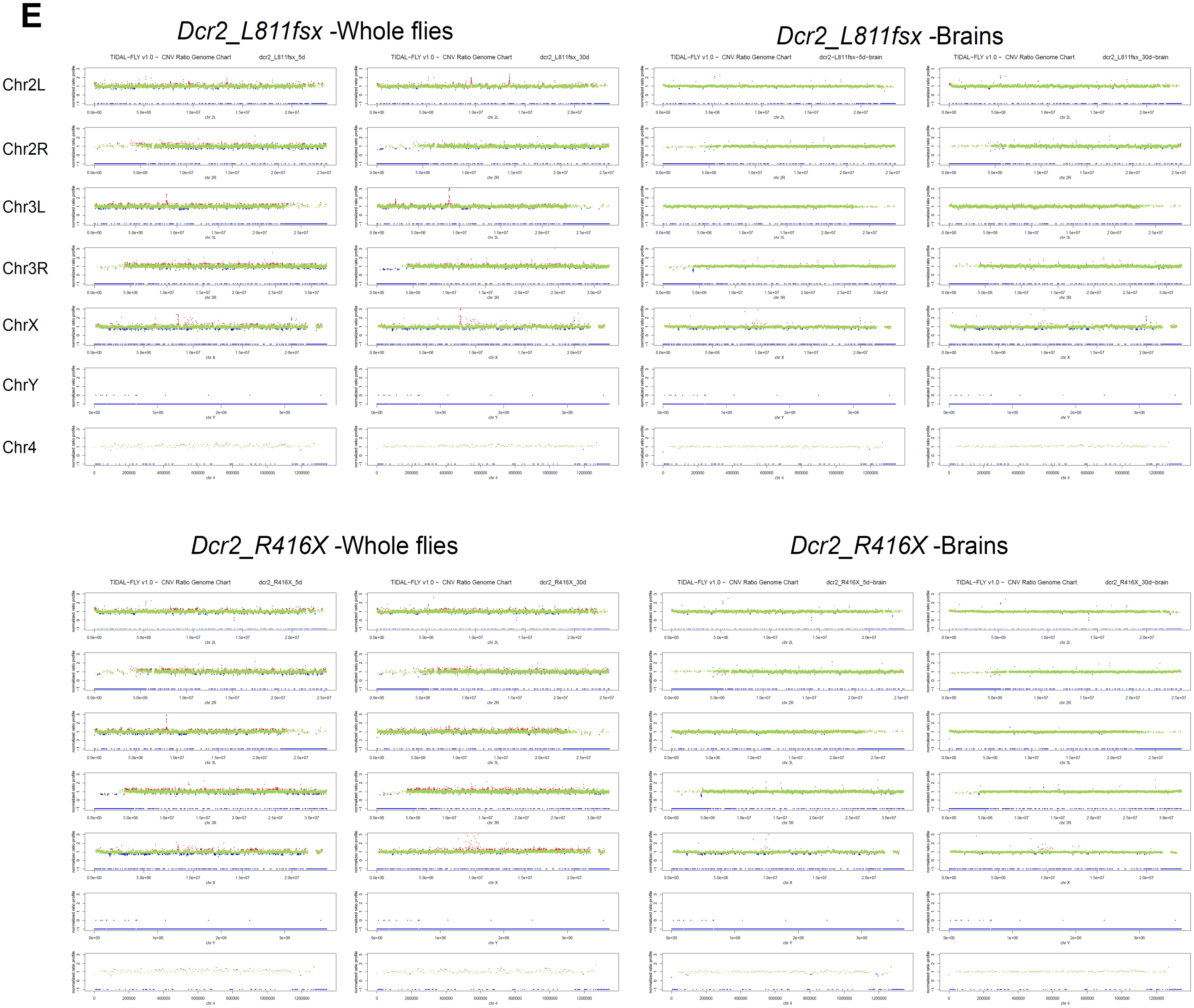

Supplement: S3 Fig — (A) Wild type fly lines libraries. (B) Aubergine and AGO3 mutants libraries. (C) Piwi mutants libraries. (D) AGO2 mutants libraries. (E) Dcr2 mutants libraries. (PDF) [file pgen.1010024.s003.pdf]
