# Supplementary material for: Transposable element landscapes in aging Drosophila
Source: PLoS Genet. 2022 Mar 3;18(3):e1010024. doi: 10.1371/journal.pgen.1010024 (PMC8893327; doi:10.1371/journal.pgen.1010024)
Supplement: S1 Table — (PDF) [file pgen.1010024.s009.pdf]

S1 Table. Statistics of the rounds of whole genome sequencing (WGS) using TIDALv1.2

Updated Sample Name	Library Size	Percent Reads Not mapped to Genome	Percent immobile gene element (IGE)	Mapped reads before downsampling	Sample Name for downstampling	Fraction number used for downsampling by Seqtk	Library Size	Percent Reads Not mapped to Genome	Mapped reads after downsampling	Absolute value of mapped reads difference between 5d and 30d	Insertion_Total	Insertion_CR>1	Insertion_CR<=1	Insertion_CR>2	Insertion_CR<=2	Insertion_Total (RPM)	Insertion_CR>1 (RPM)	Insertion_CR<=1 (RPM)	Insertion_CR>2 (RPM)	Insertion_CR<=2 (RPM)	No. TE families represented	TE Insertions norm by TE families
ISO1_5d	42,390,086	6%	6%	39,766,140	ISO1_5d_ds	0.8252	34,981,554	6%	32,814,237		40	34	6	23	17	1.22	1.04	0.18	0.70	0.52	11	3.64
ISO1_30d	35,167,146	7%	9%	32,816,328	ISO1_30d		35,167,146	7%	32,816,328	2,091	40	36	4	22	18	1.22	1.10	0.12	0.67	0.55	9	4.44
OreR_5d	36,965,737	7%	8%	34,429,887	OreR_5d		36,965,737	7%	34,429,887		698	583	115	374	324	20.27	16.93	3.34	10.86	9.41	58	12.03
OreR_30d	46,368,182	10%	6%	41,768,458	OreR_30d_ds	0.8243	38,220,319	10%	34,428,940	948	705	621	84	382	323	20.48	18.04	2.44	11.10	9.38	51	13.82
w1118_5d	34,585,247	6%	7.7%	32,506,674	w1118_5d		34,585,247	6%	32,506,674		541	483	58	301	240	16.64	14.86	1.78	9.26	7.38	52	10.40
w1118_30d	35,937,251	7%	7%	33,551,018	w1118_30d_ds	0.9689	34,818,375	7%	32,505,425	1,248	529	467	62	285	244	16.27	14.37	1.91	8.77	7.51	52	10.17
ISO1_5d_brain	34,831,057	16%	5%	29,557,635	ISO1_5d_brain		34,831,057	15%	29,557,635		36	34	2	23	13	1.22	1.15	0.07	0.78	0.44	9	4.00
ISO1_30d_brain	42,955,475	26%	40%	31,920,213	ISO1_30d_brain_ds	0.9260	39,776,599	26%	29,562,923	5,288	33	30	3	22	11	1.12	1.01	0.10	0.74	0.37	10	3.30
OreR_5d_brain	44,960,787	15%	6%	38,081,787	OreR_5d_brain_ds	0.9353	42,049,780	15%	35,617,425		638	546	92	323	315	17.91	15.33	2.58	9.07	8.84	54	11.81
OreR_30d_brain	41,578,756	14%	4%	35,616,362	OreR_30d_brain		41,578,756	14%	35,616,362	1,063	679	601	78	380	299	19.06	16.87	2.19	10.67	8.40	59	11.51
w1118_5d_brain	42,288,946	15%	6%	36,030,182	w1118_5d_brain_ds	0.7940	33,575,505	15%	28,609,117		428	382	46	192	236	14.96	13.35	1.61	6.71	8.25	50	8.56
w1118_30d_brain	38,979,690	27%	7%	28,607,194	w1118_30d_brain		38,979,690	27%	28,607,194	1,923	400	373	27	214	186	13.98	13.04	0.94	7.48	6.50	49	8.16
piwi_g1_5d	82,195,374	9%	22%	74,542,738	piwi_g1_5d_ds	0.8259	67,888,982	9%	61,567,703		566	492	74	342	224	9.19	7.99	1.20	5.55	3.64	62	9.13
piwi_g1_30d	70,142,704	12%	17%	61,566,636	piwi_g1_30d		70,142,704	12%	61,566,636	1,067	573	501	72	359	214	9.31	8.14	1.17	5.83	3.48	63	9.10
Piwi(HDR-4xP3-mCherry)_5d	68,138,461	25%	13%	51,076,590	Piwi(HDR-4xP3-mCherry)_5d_ds	0.7284	49,630,544	25%	37,208,763		572	508	64	321	251	15.37	13.65	1.72	8.63	6.75	59	9.69
Piwi(HDR-4xP3-mCherry)_30d	52,287,196	29%	8%	37,202,340	Piwi(HDR-4xP3-mCherry)_30d		52,287,196	29%	37,202,340	6,423	623	527	96	330	293	16.75	14.17	2.58	8.87	7.88	60	10.38
piwi_g1_5d_brain	38,963,764	16%	20%	32,842,557	piwi_g1_5d_brain_ds	0.9699	37,791,268	16%	31,852,748		525	485	40	358	167	16.48	15.23	1.26	11.24	5.24	56	9.38
piwi_g1_30d_brain	35,579,471	10%	7%	31,854,300	piwi_g1_30d_brain		35,579,471	10%	31,854,300	1,552	513	474	39	331	182	16.10	14.88	1.22	10.39	5.71	56	9.16
Piwi(HDR-4xP3-mCherry)_5d_brain	35,924,059	15%	11%	30,639,630	Piwi(HDR-4xP3-mCherry)_5d_brain		35,924,059	15%	30,639,630		565	509	56	333	232	18.44	16.61	1.83	10.87	7.57	56	10.09
Piwi(HDR-4xP3-mCherry)_30d_brain	39,666,916	16%	28%	33,264,676	Piwi(HDR-4xP3-mCherry)_30d_brain_ds	0.9211	36,536,806	16%	30,642,213	2,584	620	545	75	369	251	20.23	17.79	2.45	12.04	8.19	59	10.51
Ago2_2-5-14_5d	44,463,804	15%	9%	37,625,271	Ago2_2-5-14_5d_ds	0.9024	40,126,482	15%	33,955,430		537	486	51	308	229	15.81	14.31	1.50	9.07	6.74	59	9.10
Ago2_2-5-14_30d	40,686,426	17%	9%	33,952,822	Ago2_2-5-14_30d		40,686,426	17%	33,952,822	2,608	555	496	59	322	233	16.35	14.61	1.74	9.48	6.86	64	8.67
Ago2_2-16-4_5d	61,396,191	16%	4%	51,732,431	Ago2_2-16-4_5d		61,396,191	16%	51,732,431		619	459	160	261	358	11.97	8.87	3.09	5.05	6.92	65	9.52
Ago2_2-16-4_30d	62,081,936	9%	14%	56,185,394	Ago2_2-16-4_30d_ds	0.9207	57,162,528	9%	51,732,488	57	749	529	220	298	451	14.48	10.23	4.25	5.76	8.72	67	11.18
Ago2 Rescue_2-5-14_5d	44,465,825	22%	5%	34,623,003	Ago2 Rescue_2-5-14_5d		44,465,825	22%	34,623,003		242	233	9	153	89	6.99	6.73	0.26	4.42	2.57	47	5.15
Ago2 Rescue_2-5-14_30d	56,700,302	17%	5%	47,271,042	Ago2 Rescue_2-5-14_30d_ds	0.7324	41,530,738	17%	34,629,783	6,780	383	336	47	168	215	11.06	9.70	1.36	4.85	6.21	51	7.51
Ago2_2-5-14_5d_brain	41,131,381	6%	8%	38,470,181	Ago2_2-5-14_5d_brain_ds	0.8218	33,801,611	6%	31,616,235		501	442	59	289	212	15.85	13.98	1.87	9.14	6.71	57	8.79
Ago2_2-5-14_30d_brain	34,741,442	9%	5%	31,614,712	Ago2_2-5-14_30d_brain		34,741,442	9%	31,614,712	1,523	540	485	55	290	250	17.08	15.34	1.74	9.17	7.91	63	8.57
Ago2_2-16-4_5d_brain	33,172,807	18%	23%	27,042,472	Ago2_2-16-4_5d_brain		33,172,807	18%	27,042,472		505	440	65	240	265	18.67	16.27	2.40	8.87	9.80	53	9.53
Ago2_2-16-4_30d_brain	37,993,116	15%	10%	32,377,733	Ago2_2-16-4_30d_brain_ds	0.8352	31,732,611	15%	27,041,928	544	531	473	58	275	256	19.64	17.49	2.14	10.17	9.47	59	9.00
dcr2_L811fsx_5d	51,904,680	14%	9%	44,440,787	dcr2_L811fsx_5d		51,904,680	14%	44,440,787		713	527	186	271	442	16.04	11.86	4.19	6.10	9.95	67	10.64
dcr2_L811fsx_30d	64,574,190	9%	12%	58,562,333	dcr2_L811fsx_30d_ds	0.7589	49,002,795	9%	44,440,978	191	676	495	181	297	379	15.21	11.14	4.07	6.68	8.53	66	10.24
dcr2_R416X_5d	73,294,098	10%	12%	65,642,194	dcr2_R416X_5d_ds	0.7705	56,473,416	10%	50,577,704		677	533	144	321	356	13.39	10.54	2.85	6.35	7.04	67	10.10
dcr2_R416X_30d	60,048,253	16%	12%	50,578,644	dcr2_R416X_30d		60,048,253	16%	50,578,644	939	631	504	127	299	332	12.48	9.96	2.51	5.91	6.56	62	10.18
dcr2_L811fsx_5d_brain	42,466,420	32%	19%	28,809,219	dcr2_L811fsx_5d_brain		42,466,420	32%	28,809,219		630	525	105	322	308	21.87	18.22	3.64	11.18	10.69	64	9.84
dcr2_L811fsx_30d_brain	42,943,415	14%	33%	37,017,224	dcr2_L811fsx_30d_brain_ds	0.7783	33,420,377	14%	28,807,296	1,924	528	459	69	283	245	18.33	15.93	2.40	9.82	8.50	60	8.80
dcr2_R416X_5d_brain	36,338,010	9%	13%	33,005,814	dcr2_R416X_5d_brain_ds	0.8479	30,813,726	9%	27,988,415		545	487	58	294	251	19.47	17.40	2.07	10.50	8.97	58	9.40
dcr2_R416X_30d_brain	34,659,112	19%	16%	27,987,233	dcr2_R416X_30d_brain		34,659,112	19%	27,987,233	1,183	581	498	83	303	278	20.76	17.79	2.97	10.83	9.93	59	9.85
aub_g1_5d	176,183,413	28%	12%	126,177,980	aub_g1_5d_ds	0.5368	94,577,520	28%	67,720,815		213	211	2	150	63	3.15	3.12	0.03	2.21	0.93	33	6.45
aub_g1_30d	83,384,716	19%	16%	67,727,818	aub_g1_30d		83,384,716	19%	67,727,818	7,003	628	508	120	339	289	9.27	7.50	1.77	5.01	4.27	63	9.97
aub_g2_5d	132,178,268	28%	13%	94,632,767	aub_g2_5d_ds	0.5280	69,796,945	28%	49,970,844		188	180	8	122	66	3.76	3.60	0.16	2.44	1.32	36	5.22
aub_g2_30d	56,359,204	11%	12%	49,964,463	aub_g2_30d		56,359,204	11%	49,964,463	6,380	568	481	87	328	240	11.37	9.63	1.74	6.56	4.80	62	9.16
ago3_g1_5d	41,944,350	9%	10%	38,043,735	ago3_g1_5d		41,944,350	9%	38,043,735		581	459	122	284	297	15.27	12.07	3.21	7.47	7.81	57	10.19
ago3_g1_30d	55,667,008	18%	13%	45,880,748	ago3_g1_30d_ds	0.8292	46,159,249	18%	38,042,653	1,082	523	407	116	222	301	13.75	10.70	3.05	5.84	7.91	59	8.86
ago3_g2_5d	104,440,858	26%	10%	77,269,629	ago3_g2_5d_ds	0.7057	73,710,488	26%	54,542,370		304	286	18	151	153	5.57	5.24	0.33	2.77	2.81	47	6.47
ago3_g2_30d	67,373,181	19%	13%	54,531,179	ago3_g2_30d		67,373,181	19%	54,531,179	11,191	554	465	89	298	256	10.16	8.53	1.63	5.46	4.69	56	9.89
		Average IGE	12.0%				Average IGE	15.5%	Average mapped reads difference	2,852												

Inputed into Graphpad Prism

	Young	Old
Iso1-whole	3.64	4.44
OreR-whole	12.03	13.82
W1118-whole	10.40	10.17

	Young	Old
Iso1-brain	4.00	3.30
OreR-brain	11.81	11.51
W1118-brain	8.56	8.16

	Young	Old
piwi-g1-whole	9.13	9.10
piwi-HDR-whole	9.69	10.38
ago2-5-14-whole	9.10	8.67
ago2-16-4-whole	9.52	11.18
aub-g1-whole	6.45	9.97
aub-g2-whole	5.22	9.16
ago3-g1-whole	10.19	8.86
ago3-g2-whole	6.47	9.89

	Young	Old
piwi-g1-brain	9.38	9.16
piwi-HDR-brain	10.09	10.51
ago2-5-14-brain	8.79	8.57
ago2-16-4-brain	9.53	9.00
