# Supplementary material for: Transposable element landscapes in aging Drosophila
Source: PLoS Genet. 2022 Mar 3;18(3):e1010024. doi: 10.1371/journal.pgen.1010024 (PMC8893327; doi:10.1371/journal.pgen.1010024)
Supplement: S2 Table — (PDF) [file pgen.1010024.s010.pdf]

S2 Table. Oligonucleotide primers used in this study

gypsy_F	CCAGGTCGGGCTGTTATAGG
gypsy_R	GAACCGGTGTACTCAAGAGC
297_F	AAAGGGCGCTCATACAAATG
297_R	TGTGCACATAAAATGGTTCG
roo_F	CGTCTGCAATGTACTGGCTCT
roo_R	CGGCACTCCACTAACTTCTCC
I-element_F	TGAAATACGGCATACTGCCCCCA
I-element_R	GCTGATAGGGAGTCGGAGCAGATA
mdg1_F	CACATGTTCTCATTCCCAACC
mdg1_R	TTCGCTTTTTATATTTGCGCTAC
hobo-F	ACTCACACCCTACAATTTTGTGTG
hobo-R	GTGTTTAACGGTATACCCACAAGTG
jockey_F	TGCAGTTGTTTCCCCTAACC
jockey_R	AGTTGGGCAAATGCTAGTGG
blood_F	TGCCACAGTACCTGATTTCG
blood_R	GATTCGCCTTTTACGTTTGC
412_F	CACCGGTTTGGTCGAAAG
412_R	GGACATGCCTGGTATTTTGG
P-element-F	TTAATATTAGCAGCGCGAAACGTC
P-element-R	GTTGATTAACCCTTAGCATGTCCG
diver_F	TTTTTGGAGCCGACCTTATG
diver_R	GGCGTGTAATATGCGTGTTG
Het-A_F	CGCGCGGAACCCATCTTCAGA
Het-A_R	CGCCGCAGTCGTTTGGTGAGT
S-element_F	TGAAAAGCGTCATTCATTCG
S-element_R	TGTTTCTAGCGCACTCAACG
17.31_F	AGCAAACGTCTGTTGGAAGG
17.31_R	CGACAGCAAAACAACACTGC
NoF_F	AGTTGGACCTGGAATTGTGG
NoF_R	AATGCACACGGAAGAGGAAC
Idefix_F	AACAAAATCGTGGCAGGAAG
Idefix_R	TCCATTTTTCGCGTTTACTG
TAHRE_F	CTGTTGCACAAAGCCAAGAA
TAHRE_R	GTTGGTAATGTTCGCGTCCT
burdock_F	CGGTAAAATCGCTTCATGGT
burdock_R	ACGTTGCATTTCCCTGTTTC
MT_ND5_F	GCAGAAACAGGTGTAGGAGCA
MT_ND5_R	TCGAATTGGGGATGTAGCTT
Copia-F1	ATTTCCTTCTCAGAATTTGAGTG
Copia-R1	AGAATCTGACGCGCCGTAATGTC
Copia-P1	GTCGTGGTGCTGGTGTTGCAGTTG
Copia-P2	GAATAAAAAGAGTGGTATTCTCT
Copia_P8	AGGTGTGGCCATTCATATCAAATA

Copia_P9	GTGCTGGTGTTGCAGTTGAA
Copia_P10	TGTGAGTAGGTCGTGGTGCTGGTGT
Copia_P11	TTCTCTTACAATATGTTTTATGGCA
Rp49-F	ATGACCATCCGCCCAGCATAC
Rp49-R	CTGCATGAGCAGGACCTCCAG
TFIIs-F	GGATTGCGAGGCAACTTTATG
TFIIs-R	CAGCTGGGCATCGTTGATG
Paf1-F	CATCTACTACAACGAGCTAG
Paf1-R	ACGATGCTCCATGCTGTCC
Ago2-F	GTGGTTTACACGCCTCCTCA
Ago2-R	GGGTAGTTGCGACTGTGGAA
PIWI-F	TCGTACCCAATGATAACGCCGAAAG
PIWI-R	AGTCCGGACAAGGGTAGTTCGATCA
M13-F	GTAAAACGACGGCCAG
M13-R	CAGGAAACAGCTATGAC
pGL3-ScreenFW	CCTCTTCGCTATTACGCCAG
piwiPro-ScreenRV1	AAAATTTCAATCGTTGCAAGAG
Cas9-qPCR-F	CCTATCTGACATACTGAGAG
Cas9-qPCR-R	CTGACGGACTAGGGCCTTG
