# Supplementary material for: Transposable element landscapes in aging Drosophila
Source: PLoS Genet. 2022 Mar 3;18(3):e1010024. doi: 10.1371/journal.pgen.1010024 (PMC8893327; doi:10.1371/journal.pgen.1010024)
Supplement: S3 Table — (PDF) [file pgen.1010024.s011.pdf]

S3 Table. Statistics for the eccDNA sequencing.

Sample	round of sequencing	total reads	non-genome	% mapped	total normalized TE RPM	total RPM of gene locus	total RPM of 100 IGE coverage
ISO1_5d_eccDNA	10	80,124,184	14,126,324	82%	80,851	850,665	5,549
ISO1_30d_eccDNA	10	93,863,638	14,549,473	84%	65,239	1,046,201	3,991
OreR_5d_eccDNA	10	171,868,561	32,774,330	81%	58,725	517,767	6,178
OreR_30d_eccDNA	10	49,304,055	11,129,812	77%	65,780	636,615	4,476
w1118_5d_eccDNA	10	14,651,052	3,360,328	77%	81,093	685,749	6,339
w1118_30d_eccDNA	10	11,695,633	2,674,768	77%	83,355	651,368	7,614
w1118_5d_brain_eccDNA	10	13,515,783	7,702,952	43%	72,287	605,134	95,893
w1118_30d_brain_eccDNA	10	53,426,975	14,022,417	74%	52,315	472,422	92,631
OreR_5d_brain_eccDNA	10	15,769,719	4,277,015	73%	76,976	273,822	104,351
OreR_30d_brain_eccDNA	10	47,331,779	12,152,812	74%	45,203	380,664	109,388

Sample	round of sequencing	MT@chrM	Plasmid@GFP	Plasmid@pCNEPm3_6336bp	Plasmid@pGL3-DmPiwipro1	Plasmid@pGSH0_5227bps	Plasmid@pJC9F3	Plasmid@pMAXGFP	Plasmid@pUC19
ISO1_5d_eccDNA	10	470,293	713	12,086	365,543	45,981	47,612	31,858	31,823
ISO1_30d_eccDNA	10	395,508	313	3,025	81,102	10,176	10,517	7,022	7,685
OreR_5d_eccDNA	10	191,913	920	14,060	433,461	53,438	56,777	36,779	37,651
OreR_30d_eccDNA	10	210,051	272	3,017	90,584	11,190	8,602	7,787	7,967
w1118_5d_eccDNA	10	1,425,500	375	2,371	52,165	7,155	6,253	5,350	5,722
w1118_30d_eccDNA	10	101,033	173	1,977	59,690	7,483	6,669	5,077	5,355
w1118_5d_brain_eccDNA	10	771,359	3,962	21,663	425,469	61,182	46,826	47,247	49,203
w1118_30d_brain_eccDNA	10	3,451,600	2,766	31,852	857,022	111,724	81,784	77,375	81,784
OreR_5d_brain_eccDNA	10	841,007	3,144	17,386	361,569	49,613	37,647	37,759	39,766
OreR_30d_brain_eccDNA	10	2,852,930	1,517	17,308	498,556	61,312	44,585	42,908	44,359

Sample	round of sequencing	R1 Total Reads	R1 Trimmed Reads	R1 Percent	R2 Total Reads	R2 Trimmed Reads	R2 Percent	Total reads after trimming
ISO1_5d_eccDNA	10	43,314,374	4,941,273	11.40%	43,314,374	3,429,599	7.90%	78,257,876
ISO1_30d_eccDNA	10	49,299,813	3,557,839	7.20%	49,299,813	2,281,603	4.60%	92,760,184
OreR_5d_eccDNA	10	90,513,410	11,323,026	12.50%	90,513,410	11,958,631	13.20%	157,745,163
OreR_30d_eccDNA	10	27,517,013	3,828,369	13.90%	27,517,013	2,167,137	7.90%	49,038,520
w1118_5d_eccDNA	10	9,888,427	3,136,663	31.70%	9,888,427	754,235	7.60%	15,885,956
w1118_30d_eccDNA	10	7,945,211	2,600,857	32.70%	7,945,211	815,937	10.30%	12,473,628
w1118_5d_brain_eccDNA	10	12,740,321	6,516,238	51.10%	12,740,321	641,323	5.00%	18,323,081
w1118_30d_brain_eccDNA	10	30,474,488	4,559,702	15.00%	30,474,488	2,521,704	8.30%	53,867,570
OreR_5d_brain_eccDNA	10	11,768,752	4,342,637	36.90%	11,768,752	463,058	3.90%	18,731,809
OreR_30d_brain_eccDNA	10	24,416,927	1,018,864	4.20%	24,416,927	1,677,687	6.90%	46,137,303
